# Conversion of anaerobic digestates into biochar for sustainable fodder production in soilless agriculture

**DOI:** 10.1186/s40643-026-01014-7

**Published:** 2026-04-02

**Authors:** Ana L. Navas-Romero, Eliana Sánchez, Romina Zabaleta, Erick Torres, Viviana N. Fernández-Maldonado, Mathias Riveros-Gómez, Patricia Bres, Germán Mazza, M. Paula Fabani, Rosa Rodriguez

**Affiliations:** 1 Instituto de Ingeniería Química – Grupo Vinculado al PROBIEN (CONICET–UNCO), Facultad de Ingeniería, Universidad Nacional de San Juan, Av. Libertador Gral. San Martín 1109 (Oeste), 5400 San Juan, San Juan Argentina; 2 Grupo de Geobotánica y Fitogeografía – IADIZA (Instituto Argentino de Investigaciones de Zonas Áridas), CONICET–CCT Mendoza, Av. Ruiz Leal s/n, Parque General San Martín, 5500 Mendoza, Mendoza Argentina; 3 Observatorio Ambiental de San Juan, Secretaría de Ambiente y Desarrollo Sustentable, Gobierno de la Provincia de San Juan, Av. Ignacio de la Roza (Oeste) 475 (Edificio Centro Cívico), 5400 San Juan, San Juan Argentina; 4 Laboratorio de Transformación de Residuos, Instituto de Microbiología y Zoología Agrícola (IMyZA), Centro de Investigaciones en Ciencias Veterinarias y Agropecuarias (CICVyA), Instituto Nacional de Tecnología Agropecuaria (INTA), Nicolas Repetto y De los Reseros s/n, Hurlingham, 1686 Buenos Aires, Buenos Aires Argentina; 5 Instituto de Investigación y Desarrollo en Ingeniería de Procesos, Biotecnología y Energías Alternativas (PROBIEN), CONICET–Universidad Nacional del Comahue, Buenos Aires 1400, 8300 Neuquén, Neuquén Argentina; 6 Instituto de Biotecnología, Facultad de Ingeniería, Universidad Nacional de San Juan, Av. Libertador Gral. San Martín 1109 (Oeste), 5400 San Juan, San Juan Argentina

**Keywords:** Carbon-rich amendments, Soilless agriculture, Resource efficiency, Sustainable livestock feeding, Bio-waste, Circular economy

## Abstract

The valorization of anaerobic digestates through slow pyrolysis offers a sustainable pathway for agricultural systems. This study assessed the effects of digestate type (swine, cattle, and dairy) and pyrolysis temperature (400, 500, and 600 °C) on biochar properties and evaluated their impact on maize (*Zea mays*) fodder grown under hydroponic-like soilless conditions. Thirty-six treatments were evaluated in a factorial design combining digestate source, temperature, and application rate (0–6.25 g per container). Biochar from dairy digestate (BDST) pyrolyzed at 500 °C exhibited the most favorable characteristics, with high carbon content (60.4%), low electrical conductivity (≈ 916 µS cm^−1^), and improved water and nutrient retention. At an application rate of 6.25 g per container, BDST–500 also achieved the highest SPAD and dry mass values. In contrast, swine- and cattle-derived biochars presented higher ash and salinity, reducing their agronomic performance. Multivariate analysis indicated that digestate type was the main determinant of plant physiological performance, beyond the individual effects of pyrolysis temperature and application rate. Overall, dairy-derived biochar demonstrates strong potential as a functional amendment for hydroponic fodder systems and as a tool to advance circular bioeconomy practices.

**Graphical abstract:**

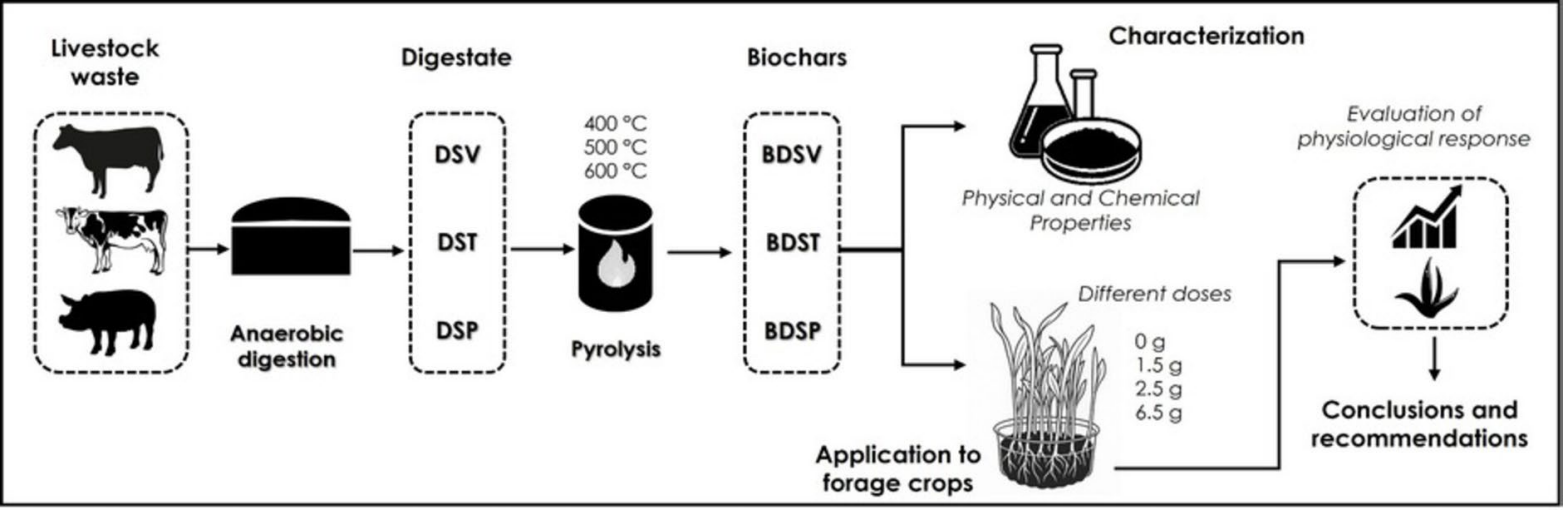

**Supplementary Information:**

The online version contains supplementary material available at 10.1186/s40643-026-01014-7.

## Introduction

The increasing global demand for food production, coupled with environmental concerns regarding waste management and resource sustainability, has prompted the exploration of alternative agricultural practices that align with circular economy principles (Lehmann and Joseph 2009). Among these strategies, the valorization of organic residues from anaerobic digestion represents a promising approach to enhancing resource efficiency. Biodigestates, the byproducts of biogas production, contain residual organic matter, nutrients, and microbial communities that can be repurposed to improve soil fertility or serve as substrates in controlled agricultural systems (Nkoa 2014). However, the direct application of biodigestates presents several challenges, including variability in composition, potential phytotoxicity, and the risk of pathogen contamination (Cucina et al. 2021). To overcome these limitations, slow pyrolysis has emerged as an effective method for transforming biodigestates into biochar, a carbon-rich material with potential applications in agriculture and environmental remediation (Xiang et al. 2020).

Several recent studies have also investigated the optimization of biochar production parameters and their influence on the resulting physicochemical properties, highlighting how temperature, residence time, and feedstock composition affect biochar yield and quality (Pawar and Panwar 2022). These works provide valuable insights into the thermochemical mechanisms governing biochar formation and further justify the temperature range selected for the present study.

**Fig. 1:**
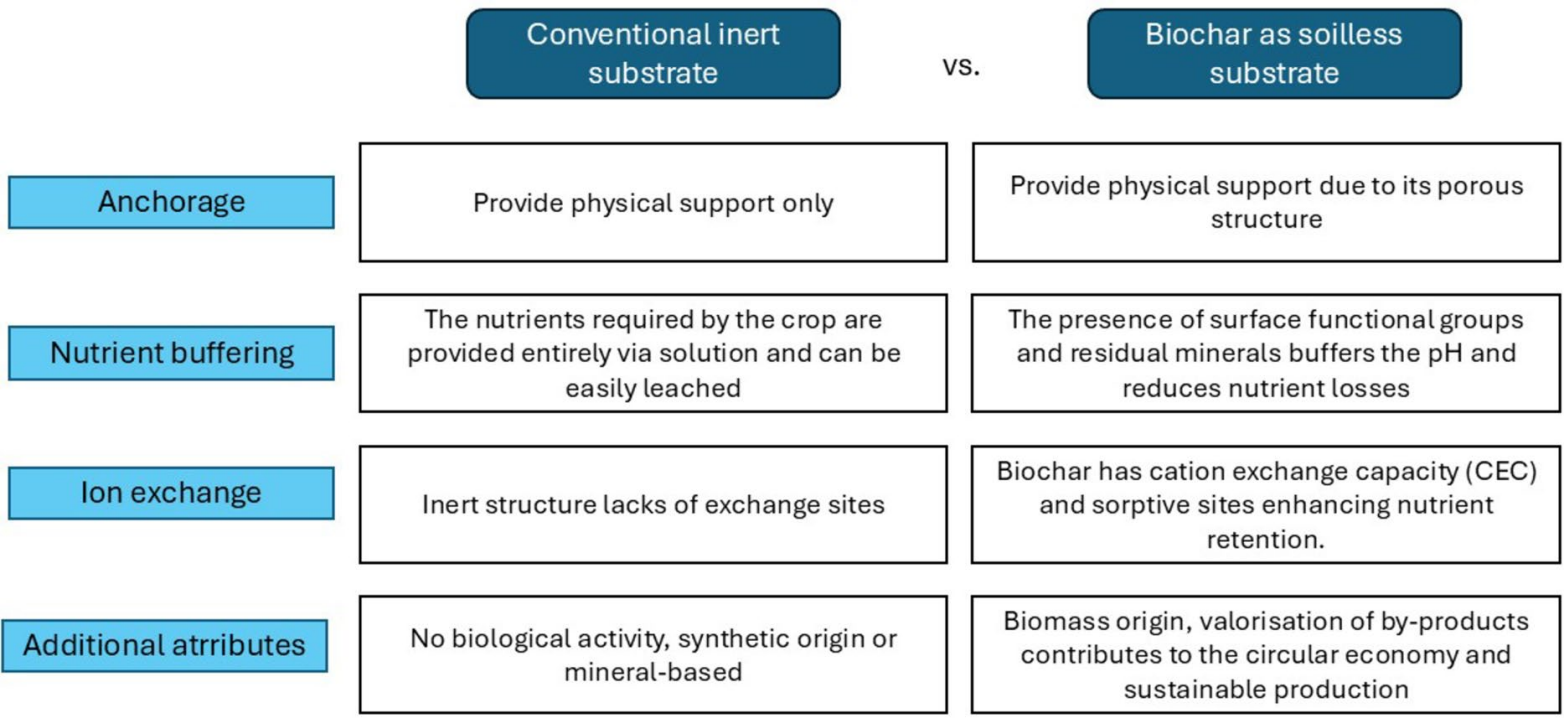
Comparison between conventional inert substrate and biochar as a soilless substrate Biochars produced at 500 °C from: **a** swine digestate (BDSP), **b** cattle digestate (BDSV), **c** and dairy digestate (BDST)

Biochar, obtained through the thermochemical decomposition of biomass under oxygen-limited conditions, has gained attention due to its ability to enhance soil structure, improve nutrient retention, and sequester carbon (Elkhlifi et al. 2023; Ullah et al. 2024). The physicochemical properties of biochar, such as porosity, surface area, and nutrient composition, are strongly influenced by the characteristics of the feedstock and the pyrolysis conditions, particularly temperature (Tomczyk et al. 2020; Sanchez et al. 2024; Zabaleta et al. 2024). Higher pyrolysis temperatures (> 500 °C) tend to increase biochar stability and carbon content while reducing volatile matter and altering nutrient availability (Pariyar et al. 2020; Tomczyk et al. 2020). Conversely, lower temperatures (300–400 °C) preserve a greater fraction of organic compounds and bioavailable nutrients, soluble minerals, and functional groups that may be more beneficial for plant nutrition and early-stage growth (Wang et al. 2020). Although traditional hydroponic systems are defined by the absence of solid substrates, modern soilless cultivation has evolved to include a broad spectrum of configurations. These include semi-hydroponic and inert media-based systems, where materials such as rockwool, perlite, coconut fiber, or even biochar serve as physical supports without contributing significant chemical reactivity (Chopra et al. 2024). Figure 1 schematically contrasts the conceptual roles of conventional inert substrates (e.g., perlite or rockwool) and reactive digestate-derived biochar, highlighting the transition from purely physical anchorage media to multifunctional substrates capable of nutrient buffering, ion exchange, and pH stabilization. The inclusion of such substrates enables better root anchorage, improved water retention, and enhanced microbial activity, while still maintaining the key principles of hydroponic and controlled-environment agriculture (Kunnen et al. 2024). Recent literature has explored the potential of integrating biochar into these systems due to its porous structure, nutrient-holding capacity, and ability to modulate rhizosphere dynamics (Chopra et al. 2024; Kunnen et al. 2024). However, the agronomic implications of using digestate-derived biochar in soilless environments remain largely unexplored, particularly in high-turnover crops like maize fodder.

Maize fodder (*Zea mays* L.) was selected as a sentinel crop due to its rapid growth, high nutrient demand, and sensitivity to variations in substrate nutrient availability, which make it a reliable indicator of nutrient use efficiency in controlled soilless systems. Its well-documented agronomic response and high biomass productivity enable the assessment of biochar performance within short cultivation cycles. Moreover, the physiological traits evaluated in maize can be extrapolated to other fast-rotating forage crops such as *Sorghum bicolor*, *Lolium multiflorum*, or *Avena sativa*, supporting the broader applicability of this model to intensive and sustainable fodder production systems (Kashyap et al. 2023).

One emerging application of biochar is its use as a growth-enhancing amendment in soilless and semi-hydroponic systems. Hydroponic fodder production, especially for livestock feed, represents a sustainable and space-efficient alternative to conventional cultivation methods. Recent studies suggest that biochar can be an effective component of hydroponic substrates, improving moisture retention, supporting microbial colonization, and providing essential nutrients for plant growth (Madani et al. 2021). However, most available studies have focused on lignocellulosic biochars (e.g., wood- or corn stover-based), with minimal data on digestate-derived biochars and their potential role in soilless systems (Loc and Do 2025).

Despite this promising outlook, research on the influence of pyrolysis temperature and feedstock origin on biochar performance in soilless systems remains limited. Particularly, there is a need to understand how these factors affect plant physiological traits, such as chlorophyll synthesis, water use efficiency, biomass accumulation, and protein content in high-turnover crops like maize fodder. Chlorophyll content, for example, is increasingly recognized as a rapid and non-destructive proxy for photosynthetic capacity and nutritive value in forage crops (Jennewein et al. 2021; Fernandes et al. 2023). On the other hand, forage protein levels are a critical parameter for livestock nutrition, often influenced by the availability and retention of nitrogen in the growing medium (Chand et al. 2022).

Although numerous studies have explored the influence of feedstock type and pyrolysis temperature on biochar properties, most research has focused on soil-based applications or lignocellulosic materials with low mineral and ash content (Ippolito et al. 2020; Das et al. 2021; Pariyar et al. 2020). These studies demonstrated that temperature-induced variations affect attributes such as porosity, ash content, nutrient retention, and carbon stability, which ultimately determine biochar’s suitability for different agricultural applications. However, the functional performance of digestate-derived biochars in soilless or hydroponic systems remains largely unknown (Castro-Herrera et al. 2022). The lack of systematic evaluations integrating thermochemical processing conditions, feedstock origin, and application rate limits our understanding of how these factors jointly determine carbon stability, nutrient release dynamics, and plant physiological performance in nutrient-limited environments. Moreover, the relationships between biochar physicochemical attributes, particularly electrical conductivity (EC), ash composition, and organic matter (OM) content, and key physiological indicators such as chlorophyll synthesis, biomass accumulation, and crude protein formation have not been clearly established (Chopra et al. 2024). In this context, the combined effects of feedstock origin, pyrolysis temperature, and application dose on plant physiological responses under hydroponic conditions remain poorly understood, highlighting the need for comprehensive studies addressing this complex interaction.

Therefore, the present work offers a systematic evaluation of the impact of pyrolysis temperature on the agronomic properties of biochar produced from three different biodigestates: cattle (*Bos taurus*), swine (*Sus scrofa domesticus*), and mixed dairy-farm residues. Biochars were generated at 400, 500, and 600 °C, and were then characterized in terms of elemental carbon (C) content, volatile matter (VM) content, ash chemistry, porosity, pH, and cation-exchange capacity. These physicochemical data are coupled with a hydroponic bioassay using maize (*Zea mays*) fodder to quantify fresh and dry biomass accumulation, chlorophyll (SPAD) dynamics, tissue moisture, and crude protein concentration at various application rates. This was achieved by integrating the origin of the feedstock, the thermal regime, and the dosage into a single factorial design. The study aims to: (i) identify the optimal pyrolysis temperature range that balances carbon stability and nutrient availability; (ii) determine the most efficient biochar treatments for hydroponic systems by integrating crop productivity and physiological efficiency; and (iii) evaluate the effect of different doses of the most efficient biochar treatments on the nitrogen and crude protein content of forage to optimize its nutritional value and applicability in soilless agricultural systems. The resulting insights not only advance the fundamental understanding of feedstock and temperature-dependent biochar behavior but also generate actionable guidelines for the valorization of anaerobic digestion residues as high-value inputs in hydroponic forage systems, thereby reinforcing circular bioeconomy objectives and informing future climate-smart livestock production.

## Methods

### Collection and preparation of biodigestates

Three types of solid digestates were collected from large-scale biogas plants in Buenos Aires Province, Argentina: DSP (swine digestate), DSV (cattle digestate), and DST (dairy digestate). Each digestate originated from a distinct processing facility with specific feedstock compositions and operating conditions: DSP was sourced from a facility that treats swine effluent and is maintained within a temperature range of 38–42 °C (35°22ʹ59.75ʺS; 59°26ʹ33.21ʺW). DSV was obtained from a biogas plant that processes feedlot cattle manure (60%) and forage (40%), operating at a temperature range of 38–42 °C (37°54ʹ8.62ʺS; 67°42ʹ58.24ʺW). DST was collected from a plant processing dairy cattle manure (90%), along with discarded feed, spoiled silage (9%), and glycerin (1%), operating at temperatures between 34 and 40 °C (34°14ʹ10.91ʺS; 62°2ʹ49.62ʺW). All samples were initially oven-dried at 50 °C for one week, then ground using a TecnoDalvo TDMC mill. The initial moisture content of the digestates ranged between 60 and 80%, and after drying, a final moisture content below 6% was achieved, consistent with the requirements for subsequent slow-pyrolysis processing (Maqhuzu et al. 2021; Capossio et al. 2022). The dried material was sieved through a 2 mm mesh to ensure homogeneity for subsequent chemical and germination assays and was stored in sealed polyethylene bags under dry conditions at room temperature until further use.

### Slow pyrolysis and biochar production

The dried digestate samples underwent slow pyrolysis in a cylindrical pyrolysis reactor under an inert nitrogen atmosphere (5 L h^−1^). The process was carried out at three target temperatures: 400, 500, and 600 °C, with a residence time of 2 h for each temperature. The samples were introduced into the reactor only after the system reached the selected temperature to ensure consistent processing conditions. The pyrolysis reactor, as described by Rodriguez-Ortiz et al. (2020), had a feedstock capacity of 4 kg and was heated using an electric resistance system with an approximate power of 2000 W. To ensure temperature stability, the reactor temperature was monitored with a type-K thermocouple placed adjacent to the sample bed, confirming that the setpoint (400, 500, or 600 °C) was maintained within ± 3 °C throughout the 2 h residence time. Each run was preceded by a 30-min N_2_ purge, and oxygen concentration at the reactor outlet was measured (< 1.0%) to verify leak-free and inert conditions. These checks confirm that the intended pyrolysis temperatures were consistently achieved, although dynamic heating-rate data were not recorded during the sample treatment stage.

**Fig. 2:**
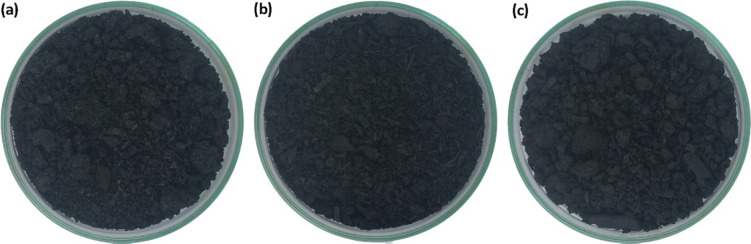
Biochars produced at 500 °C from: **a** swine digestate (BDSP), **b** cattle digestate (BDSV), **c** and dairy digestate (BDST)

The heating rate was set to approximately 10 °C per minute to ensure controlled thermal decomposition. This experimental design aimed to isolate the influence of pyrolysis temperature on biochar characteristics while maintaining uniform feedstock conditions across all trials. The selected pyrolysis temperatures (400, 500, and 600 °C) were chosen to represent low-, medium-, and high-carbonization regimes typically employed in slow pyrolysis of agricultural residues. Lower temperatures (< 400 °C) often yield biochars with unstable organic fractions and high volatile content, whereas temperatures above 600 °C substantially reduce nutrient retention and surface functionality. This range, therefore, captures the key transition between biochars optimized for nutrient availability and those optimized for structural stability (Pariyar et al. 2020; Tomczyk et al. 2020). Figure 2 shows representative images of the biochar produced at 500 °C.

### Characterization of Biochars

The biochars obtained through slow pyrolysis were characterized by conducting proximate and elemental analyses. First, a proximate analysis was performed on each biochar sample, BDSP (biochar from swine digestate), BDSV (biochar from cattle digestate), and BDST (biochar from dairy digestate), at the three studied temperatures. Ash content, VM, and fixed carbon (FC) were determined according to ASTM standards (ASTM 2001, 2006). Elemental analysis was performed using an elemental analyzer (AuroEA3000, Elementar Analysensysteme GmbH, Langenselbold, Germany). Additionally, elemental composition was determined by measuring carbon (C), hydrogen (H), and oxygen (O) contents using an elemental analyzer (AuroEA3000, EuroVector S.R.L., Redavalle, Italy). The yield of each biochar was calculated as the ratio between its weight and the initial biomass weight. The determination of OM (organic matter) content was carried out according to the methodology described in UNE-EN 13039 (AENOR 2001). The moisture content was determined using an infrared moisture analyzer (Radwag PMR50, Radom, Poland), and results were expressed as a percentage. Moreover, pH and EC were measured for each sample in a suspension of biochar in distilled water at a ratio of 1:10 (w v^−1^) according to the methodology proposed by Belda et al. (2016), using a digital pH meter (Adwa AD1000, Adwa Instruments Kft, Hungary) and a conductivity meter (EC-214, Hanna Instruments, Italy), respectively. All parameters were measured in triplicate to ensure analytical reproducibility.

### Seed selection and pretreatment

To produce hydroponic green forage (HGF), maize grains (*Zea mays*) were selected based on quality criteria such as harvest age, collection time, germination capacity, and cleanliness. Maize was chosen over other forage options due to its high germination rate, rapid biomass production, and significant crude protein and energy content, which make it highly suitable for hydroponic cultivation systems, especially in arid and semi-arid regions (Singh et al. 2024). Its relevance in local livestock feeding practices and adaptability to controlled environments further support its selection. To ensure uniformity of the seeds, the grains were dry screened to remove any broken, burnt, or impure seeds. The seeds then underwent a two-stage cleaning and disinfection process to minimize the risk of contamination and fungal proliferation. First, the grains were washed with 800 cm^3^ of water per kilogram of seed to remove surface dirt. Then, they were disinfected using a 1% sodium hypochlorite solution (diluted from a 5% stock solution) for a maximum immersion time of 2 min. Finally, the seeds were thoroughly rinsed with distilled water to remove any residual hypochlorite before sowing to prevent chemical stress during germination.

### Experimental design

**Fig. 3:**
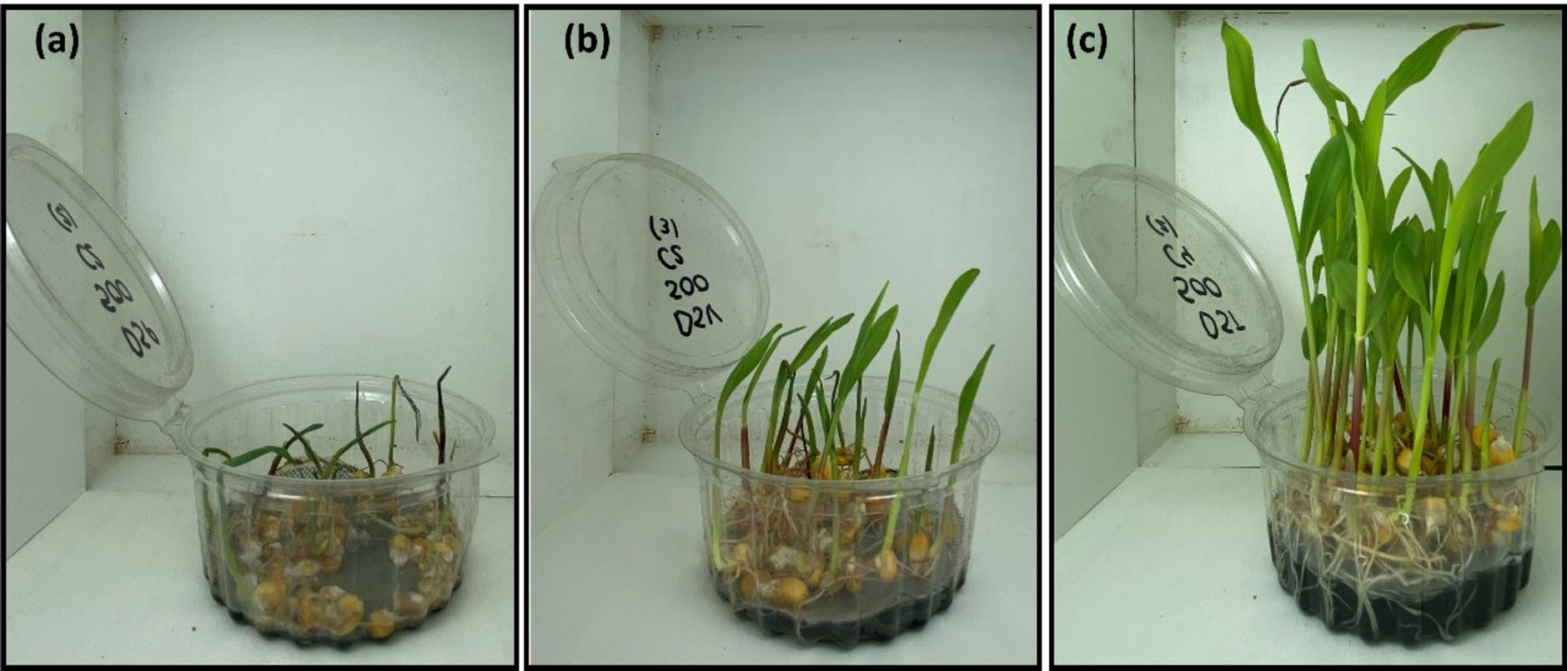
Visual response of hydroponic maize fodder to different biochar treatments derived from anaerobic digestates. Representative growth after 8 days under biochar from **a** swine digestate (BDSP), **b** cattle digestate (BDSV), and **c** dairy digestate (BDST) biochar treatments, respectively, at 500 °C and 6.25 g per container dose

**Fig. 4:**
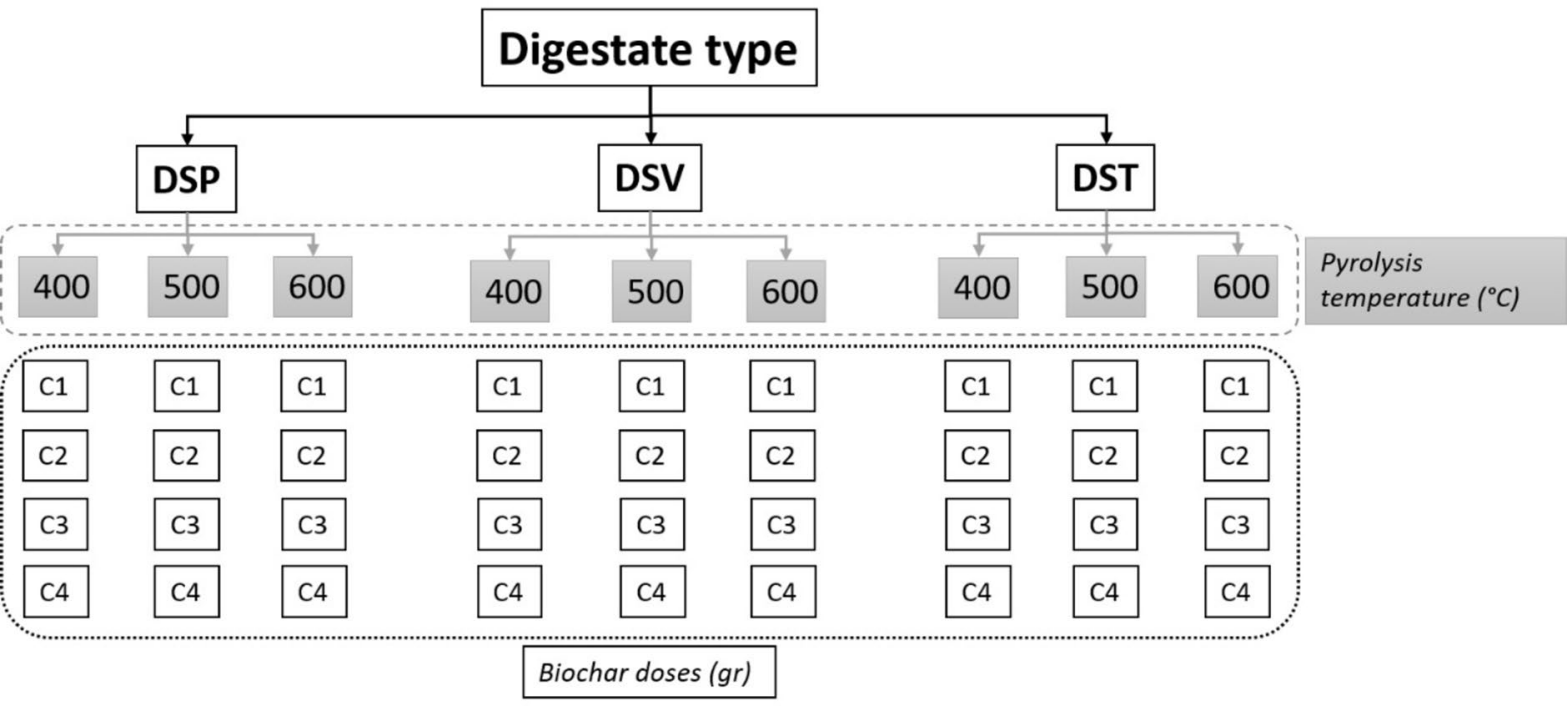
Description of treatments applied. Detailed treatment coding. BDSP, BDSV, and BDST: Biochar from swine, cattle, and dairy digestates, respectively. C1: 0 g, C2: 1.25 g, C3: 2.50 g, C4: 6.25 g per container

A 3 × 3 × 4 factorial experimental design was implemented to evaluate the agronomic response of hydroponic maize fodder to biochars derived from different digestates, processed at multiple pyrolysis temperatures, and applied at varying doses. The study comprised a total of 36 treatments, resulting from the combination of three experimental factors: digestate type, pyrolysis temperature, and biochar application dose. Digestate type (3 levels): BDSP, BDSV, BDST. Pyrolysis temperature (3 levels): 400, 500, and 600 °C. Biochar dose (4 levels): C1: 0 g per container (control), C2: 1.25 g per container, C3: 2.50 g per container, C4: 6.25 g per container. The selection of the four biochar doses was based on previous literature, particularly Solaiman et al. (2012), who reported these rates as optimal to evaluate dose-dependent effects on plant growth without inducing toxicity or nutrient imbalances. This range allows assessing the low to high application rates commonly used in hydroponic and soilless systems. Each treatment was applied in a hydroponic maize (*Zea mays*) fodder production system under controlled environmental conditions. Biochar for each treatment was incorporated into an inert substrate used for the cultivation of soilless fodder. All biochars were used as produced, without pre-rinsing or conditioning, to evaluate their agronomic performance under realistic conditions of direct application. This approach allowed assessing potential short-term effects associated with residual alkalinity and soluble salts inherent to each feedstock. Experiments were conducted in transparent polypropylene containers, with each one assigned to a single treatment (Fig. 3). To prevent root penetration into the biochar layer, each container was lined with two plastic meshes of differing mesh sizes. After preparing the substrate, 50 maize (*Zea mays*) seeds were evenly distributed between the containers. The containers were irrigated with tap water until the substrate was fully moistened and kept in complete darkness for 48 h, or until the seed pericarp displayed a reddish hue, indicative of germination onset. The pH of the nutrient solution varied between 5.5 and 6.5, which is the recommended range for optimal nutrient uptake in hydroponic maize cultivation. The nutrient solution was prepared and applied once at the beginning of the experiment and was not replaced or refreshed during the 8-day growth period. This design was intentional to evaluate potential short-term shifts in solution chemistry attributable to biochar salinity and alkalinity under closed-system conditions. pH and EC of the nutrient solution were measured at day 0 (after 45 min equilibration with the biochar dose) and again at day 8. Thereafter, they were maintained under controlled environmental conditions with a 12 h light / 12 h dark photoperiod using natural light augmented with full-spectrum LED lamps (220 µmol m⁻^2^ s⁻^1^). The experiment was carried out under stable environmental conditions. Temperature was maintained at 25 ± 2 °C with a relative humidity of 60–70%. The containers were irrigated twice daily to maintain consistent moisture throughout the experiment. Each irrigation provided approximately 20–25 mL of tap water per container per day, divided into two equal doses to ensure uniform moisture in the substrate. Watering was done manually to avoid overflow or leaching. The experiment was designed using a completely randomized design with three replicates per treatment, totaling 108 experimental units. After 8 days, the following plant variables were assessed: fresh weight, dry weight, chlorophyll content (SPAD index), and tissue moisture percentage. The factorial design scheme of the work can be observed in more detail in Fig. 4.

Although this setup may differ from traditional liquid-flow hydroponic systems, the experimental design represents a valid soilless cultivation model widely used in fodder production, particularly in regions where high-tech systems are unavailable. Moreover, the goal was not to emulate advanced recirculating hydroponics but to test the functional compatibility of digestate-derived biochars in nutrient-limited, water-controlled root environments.

### Growth monitoring and harvesting

Seed germination was observed within 24 h. After 8 days of growth, a uniform green carpet approximately 24 cm high with root systems averaging 6 cm long had developed. The optimal harvest window was set at 14 days, beyond which the nutritional value of the forage declined, with peak nutritional quality occurring between days 6 and 8. On day 8, the intact maize forage mat was harvested. Fresh weight was recorded. Chlorophyll content was estimated using a portable SPAD chlorophyll meter (CM-B, Biobase, Shandong, China). Measurements were conducted on the expanded leaf of each plant, placing the SPAD meter on the middle portion of each leaf to ensure consistency across samples. To minimize variability caused by light intensity, all readings were taken under stable ambient conditions and at similar times of day. The SPAD meter was calibrated in accordance with the instruction manual before each measurement session. High SPAD readings indicate increased leaf chlorophyll content and are closely associated with high nitrogen availability in plant tissues. The meter consists of two arms that open and close in the form of a clamp. The front side of the maize leaves was placed above the measuring chamber of the SPAD meter, and measurements were taken by closing both arms. Ten readings were taken in each test, and the average of these readings was calculated. Moisture content was determined using a halogen moisture analyzer (OHAUS model MB35 HALOGEN, Parsippany, NJ, USA) on a fresh weight basis, following standard AOAC methods (AOAC 2010). Subsequently, samples were oven-dried at 105 °C to obtain dry weight and milled for proximate analysis. Dry matter was determined according to standard AOAC methods (AOAC 2010).

### Determination of crude protein and nitrogen content

After completing the full agronomic assessment of all treatments, including elemental and proximate characterization of the biochars and their impact on hydroponic maize (*Zea mays*) performance (fresh weight, dry biomass, moisture content, and SPAD chlorophyll index), one treatment was selected for further nutritional analysis. The choice was made retrospectively, based on its combination of favorable physicochemical properties (e.g., moderate ash content, low EC) and its superior effects on plant biomass and chlorophyll content. Nitrogen content in the harvested maize fodder was determined using the Kjeldahl method (AOAC 2010) with a digestion system (Velp Scientifica DK20, Italy) and a distillation unit (Velp UDK 139, Italy). Crude protein content was then calculated using a conversion factor (N × 6.25) based on nitrogen content determined by the Kjeldahl method. Protein values were expressed on a dry matter basis. This analysis was restricted to the treatment with the most favorable agronomic and chemical profile to evaluate its nutritive potential under optimal conditions, rather than comparing all treatments irrespective of their agronomic viability*.* Based on the results obtained, the treatment BDST-500 was selected for nutritional analysis, as it demonstrated the best agronomic performance among all biochar and temperature combinations.

### Statistical analysis

All treatments were conducted in triplicate, and the data are reported as mean ± standard deviation. Biochar characterization data were analyzed by ANOVA, and significant differences between mean values were determined by the Tukey test (*p* < 0.05). Statistical independence of the data was assessed, and normality was verified using the Kolmogorov–Smirnov test. Homoscedasticity was verified using the Levene test. All analyses were performed considering α = 0.05. Data analysis was performed using Infostat statistical software, version 1.1 (InfoStat Group, Facultad de Ciencias Agropecuarias, Universidad Nacional de Córdoba, Argentina)*.*

To further explore integrated response patterns and identify the most effective treatments, a Principal Component Analysis (PCA) was conducted. This multivariate analysis enabled the reduction of dataset dimensionality by evaluating the covariation among dry biomass, chlorophyll content, and tissue water content across different combinations of biochar type, pyrolysis temperature, and application dose. Additionally, complementary physiological parameters were incorporated, such as the efficiency of dry biomass conversion relative to retained moisture, to evaluate the capacity of the substrate to convert water into functional plant tissue. This integrative approach allowed us to evaluate not only absolute productivity but also the efficiency of resource use, providing a more holistic view of treatment performance under hydroponic conditions.

Furthermore, a mixed-factorial multivariate analysis of variance (MANOVA) was conducted to evaluate the effects of BDSP, BDST, BDSV, pyrolysis temperature (400, 500, and 600 °C), and biochar application dose (0, 1.25, 2.5 and 6.5 g per container) on four agronomic response variables: fresh weight, dry weight, moisture content, and SPAD chlorophyll index. All three factors were treated as fixed, and the model included the following interaction terms: (i) digestate type × pyrolysis temperature, (ii) digestate type × biochar dose, (iii) pyrolysis temperature × biochar dose, and (iv) digestate type × pyrolysis temperature × biochar dose. Multivariate significance was assessed using Wilks’ Lambda (*p* < 0.05). Where significant multivariate effects were found, follow-up univariate ANOVAs were conducted for each dependent variable, and mean comparisons were performed using Tukey’s Honestly Significant Difference (HSD) test at a 5% significance level (α = 0.05). Multivariate normality was assessed using Mardia’s skewness and kurtosis test, considering the dependent variables fresh weight, dry weight, moisture content, and SPAD chlorophyll index. Homogeneity of the variance–covariance matrices was assessed using Box’s M test, considering both the main effects and their interactions (Table S1, Supplementary Information). Univariate normality of the residuals was checked using the Shapiro–Wilk test, applied to the residuals of the linear models fitted for each response variable. Homogeneity of variances was verified using Levene’s test (Table S2, Supplementary Information). In all cases, a significance level of α = 0.05 was considered. Independence of observations was ensured through the random assignment of treatments to experimental units. Effect size was estimated using partial eta squared (η^2^_p_) for each dependent variable and each main factor (digestate type, pyrolysis temperature, and biochar doses), as well as for their interactions. The 95% confidence intervals were calculated. All statistical analyses were carried out using SPSS Statistics v17 and R-Studio v2025.09.1.

In addition, to determine the nutritional quality of the forage based on nitrogen and crude protein content in the treatment with the most favorable agronomic and chemical profile, an ANOVA was performed, considering the applied dose (C1, C2, C3, and C4) as the single factor. Differences between means were assessed using Tukey’s HSD test at a significance level of *p* < 0.05. This statistical analysis allowed for the identification of the optimal dose of the treatment in terms of nutritional contribution. Planarity analysis indicated that both nitrogen content and crude protein content did not show significant curvature with the applied doses (*p* > 0.05).

## Results and discussion

### Biochar characterization

The physicochemical properties of BDSP, BDSV, and BDST at pyrolysis temperatures of 400, 500, and 600 °C are summarized in Table 1. Slow-pyrolysis converted the three solid digestates into highly alkaline (pH 9.3–10.9), carbon-rich biochars with proximate and elemental properties significantly influenced by both feedstock and temperature (Table 1). Moisture content was low for all biochars (< 5%), with the lowest average observed in BDST–500 (2.9 ± 0.3%), which was significantly lower than for BDSP–600 and BDST–600 (*p* < 0.05), indicating higher thermal dryness at an intermediate temperature. VM decreased with increasing pyrolysis temperature, with BDST-500 showing one of the lowest VM values (16.0 ± 0.3%). The reduced VM content enhances stability and minimizes the leaching of phytotoxic compounds. High-volatility biochar derivatives often release labile organic fractions that can inhibit plant growth (Tomczyk et al. 2020). Similar VM decreases have been described between 300 °C and 500 °C in manure-based biochars, which are considered a prerequisite for long-term structural stability (Wang et al. 2020). Ash content increased with pyrolysis temperature in most cases, reaching a peak in BDSP–500 (35.5 ± 0.04%) while BDST–400 (29.1 ± 0.5%) and 500 (30.1 ± 0.1%) exhibited significantly lower ash content, suggesting an increase in inorganic residues in BDSP. Although ashes provide essential macronutrients to plants (Ca, Mg, K, P), they also increase EC and can induce osmotic stress, especially in soilless crops (Grafmüller et al. 2022). The BDST–500 treatment had significantly lower ash content than the other digestate treatments at 500 °C, reducing the presence of undesirable salts. Its low EC (< 1000 µS cm^−1^) supports favorable conditions for root development. High-salinity biochars (such as BDSV–500) consistently impair plant growth due to osmotic stress (Murtaza et al. 2024). Total C was highest in BDST–500 (60.4 ± 0.4%), significantly greater than BDSV–500 (57.04 ± 0.21%) and BDSP–500 (53.5 ± 0.3%). This elevated C percentage of BDST–500 is indicative of greater aromatic and stable carbon structures, essential for nutrient retention and long-term efficacy in agronomic systems (Novak et al. 2021).

**Table 1:** Main properties of the studied biochar. Values are means ± standard deviation. ANOVA

	BDSP			BDSV			BDST		
	400 °C	500 °C	600 °C	400 °C	500 °C	600 °C	400 °C	500 °C	600 °C
Moisture (%)	3.0 ± 0.1^b^	3.3 ± 0.3^b^	4.4 ± 0.4^a^	3.9 ± 0.2^ab^	3.0 ± 0.2^b^	3.3 ± 0.2^b^	3.9 ± 0.6^ab^	3.0 ± 0.3^b^	4.4 ± 0.4^a^
Ash (%)	31.3 ± 0.6^c^	35.5 ± 0.1^a^	33.4 ± 0.1^b^	31.8 ± 0.5^c^	33.3 ± 0.2^b^	33.6 ± 0.1^b^	29.1 ± 0.5^d^	30.1 ± 0.1^d^	32.0 ± 0.5^c^
VM (%)	26.3 ± 0.4^a^	19.5 ± 0.2^d^	21.7 ± 0.1^c^	23.5 ± 0.8^b^	16.9 ± 0.2^e^	19.8 ± 0.8^d^	20.0 ± 0.6^d^	16.0 ± 0.3^e^	20.7 ± 0.1^ cd^
FC (%)	39.4 ± 0.1^f^	41.7 ± 0.4^d^	40.6 ± 0.1^e^	40.8 ± 0.1^de^	46.8 ± 0.1^b^	43.3 ± 0.5^c^	47.0 ± 0.5^b^	50.9 ± 0.4^a^	42.9 ± 0.2c
C (%)	54.1 ± 0.2^ fg^	53.2 ± 0.3^ g^	54.0 ± 0.3^ fg^	54.7 ± 0.1^ef^	57.0 ± 0.2^c^	55.2 ± 0.4^de^	59.0 ± 0.3^b^	60.4 ± 0.4^a^	55.7 ± 0.1^d^
H (%)	2.5 ± 0.3^a^	2.0 ± 0.1^ab^	2.2 ± 0.2^ab^	2.3 ± 0.3^ab^	1.9 ± 0.2^ab^	2.0 ± 0.1^ab^	2.1 ± 0.3^ab^	1.8 ± 0.1^b^	2.1 ± 0.1^ab^
O (%)	10.9 ± 0.3^a^	7.4 ± 0.2^e^	8.6 ± 0.1^c^	9.6 ± 0.3^b^	6.3 ± 0.4^f^	7.6 ± 0.1^de^	8.2 ± 0.4^cde^	6.1 ± 0.4^f^	8.3 ± 0.2^ cd^
Other elements (%) (*)	1.2 ± 0.1^a^	1.6 ± 0.4^a^	1.9 ± 0.1^a^	1.6 ± 0.3^a^	1.6 ± 0.4^a^	1.6 ± 0.3^a^	1.6 ± 0.1^a^	1.5 ± 0.3^a^	1.9 ± 0.2^a^
H/C	0.55 ± 0.06^a^	0.45 ± 0.01^abc^	0.48 ± 0.05^abc^	0.50 ± 0.07^ab^	0.39 ± 0.03^bc^	0.44 ± 0.02^abc^	0.43 ± 0.07^abc^	0.37 ± 0.03^c^	0.45 ± 0.02^abc^
O/C	0.15 ± 0.01^a^	0.10 ± 0.01^d^	0.12 ± 0.01^c^	0.13 ± 0.01^b^	0.08 ± 0.01^e^	0.10 ± 0.01^d^	0.10 ± 0.01^d^	0.08 ± 0.01^e^	0.11 ± 0.01^ cd^
Yield (%)	47.7 ± 2.2^ab^	43.9 ± 2.1^b^	42.1 ± 3.3^b^	51.3 ± 2.5^a^	47.3 ± 1.6^ab^	45.3 ± 2.9^ab^	48.6 ± 1.5^ab^	44.4 ± 2.8^b^	43.7 ± 1.6^b^
OM (%)	68.6 ± 0.4^b^	66.0 ± 0.1^e^	67.1 ± 0.5^ cd^	68.0 ± 0.5^bc^	64.9 ± 0.3^f^	66.2 ± 0.1^de^	71.6 ± 0.5^a^	68.9 ± 0.1^b^	67.6 ± 0.1^c^
pH	9.9 ± 0.1^e^	10.7 ± 0.1^bc^	10.3 ± 0.1^d^	10.3 ± 0.1^d^	10.9 ± 0.1^a^	10.3 ± 0.1^d^	10.6 ± 0.1^c^	10.7 ± 0.1^ab^	9.3 ± 0.1^f^
EC (µS cm^−1^)	1176 ± 228^bc^	1135 ± 132^bc^	1204 ± 9^b^	2140 ± 14^a^	2360 ± 354^a^	2250 ± 156^a^	746 ± 70^c^	916 ± 18^bc^	839 ± 10^bc^

Values followed by similar letters are not significantly different between treatments (Tukey test, *p* < 0.05). Letters (a, b, c, d, e, f, g) represent the significance levels among the means of the different groups or treatments. BDSP (biochar from swine); BDSV (biochar from cattle); BDST (biochar from dairy); VM (volatile matter); FC (fixed carbon); C (carbon content); H (hydrogen content); O (oxygen content); OM (organic matter); EC (electrical conductivity)

* Mainly N, S, and other minerals present in ashes

H/C and O/C ratios (Fig. S1, Supplementary Information) indicated that BDST–500 exhibited higher aromaticity and lower functional-group density. These values fall below the commonly accepted thresholds for biochar stability and aromaticity (H/C < 0.6; O/C < 0.2), indicating that BDST–500 has the most recalcitrant carbon structure of all the samples and is therefore ideal for long-term persistence in agronomic systems (Novak et al. 2021). By contrast, BDSP–400 displayed the highest H/C and O/C ratios (0.55 and 0.15, respectively), indicating less carbonization and higher concentrations of reactive surface groups. These molar ratios confirm the combined effect of digestate type and pyrolysis temperature on the structural maturity and oxidation level of the resulting biochar.

The biochar yields obtained from the three digestates (BDSP, BDSV, and BDST) showed a consistent decrease with increasing pyrolysis temperature, reflecting the expected thermochemical degradation behavior of organic matter. Across treatments, yields declined from approximately 51–48% at 400 °C to 45–42% at 600 °C, with an average reduction of 5–6%. This reduction is attributed to enhanced devolatilization and the conversion of organic matter into gaseous and condensable phases as temperature rises, thereby decreasing the solid carbonaceous residue retained as biochar (Wang et al. 2020). Among feedstocks, BDSV exhibited the highest yields at all temperatures (51.3%, 47.3%, and 45.3% at 400, 500, and 600 °C), suggesting a higher proportion of thermally stable inorganic components, whereas BDSP and BDST displayed slightly lower yet comparable yields (47.7–42.1% and 48.6–43.7%, respectively). These relatively high conversion efficiencies indicate that the slow pyrolysis conditions applied were suitable for producing nutrient-rich biochars while maintaining favorable material recovery rates.

OM content was generally high in all treatments, with treatment BDST–400 showing the highest values (71.6 ± 0.5%), which was significantly greater than those of the other treatments. BDSV–500 presented the lowest OM content (64.9 ± 0.3%). However, when the pyrolysis treatments at 500 °C were compared, the OM content was highest in BDST–500 (68.9 ± 0.1%), followed by BDSP–500 (66.0 ± 0.1%) and BDSV–500.

All of the biochars displayed an alkaline pH (> 9). The treatment BDST–500 had a pH of 10.7 ± 0.1, significantly higher than BDSP–400 and BDST–600, yet still within a range compatible with soilless nutrient systems. Although high pH can limit nutrient availability, moderate alkalinity supports nutrient buffering without impairing uptake, unlike the more extreme pH values, which can destabilize ionic equilibria in nutrient solutions (Tusar et al. 2023). The lowest EC value was observed at BDST–400, while the highest was recorded at BDSV–500. However, when the treatments at a temperature of 500 °C were compared, EC was markedly lower in BDST–500 (916 ± 18 µS cm^−1^) and BDST–400 (746 ± 70 µS cm^−1^) than in BDSP–500 (1135 ± 132 µS cm^−1^) and especially BDSV–500 (2360 ± 354 µS cm^−1^), indicating reduced salinity.

Studies have reported that biochar addition can stabilize nutrient release and improve plant nutrient uptake. For example, biochar reduced N leaching while enhancing P availability in swine manure-derived biochars (Banik et al. 2021; Wang et al. 2022). The treatment BDST–500 could provide a stable matrix for nutrient adsorption and controlled release due to its high C and moderate mineral content. Increasing the set-point to 600 °C marginally reduced VM and H/C but did not result in any further reduction in EC, but led to an increase in ash in BDST and BDSP. Meta-analyses show similar diminishing returns above 550 °C, where further aromatization no longer compensates for nutrient loss and greater energy input (Nkomo et al. 2021; Li et al. 2023). Comparing these digestate chars with lignocellulosic biochars (e.g., corn-stover or wood at 500 °C, typically 75–85% C, ash < 5%, EC < 300 µS cm^−1^) underscores their distinct niche: digestate chars provide a greater quantity of intrinsic nutrients, but require more rigorous EC management. The dairy-derived BDST–500 appears to strike the best balance: its EC falls within the recommended range for nutrient film and floating-raft hydroponics (< 1200 µS cm^−1^), while its ash confers macro- and micronutrients lacking in lignocellulosic chars.

In summary, the quality of the biochar was more influenced by the chemical composition of the feedstock than by the pyrolysis temperature. DST pyrolysis produces biochar with medium-ash content, low salinity, and carbon density, properties that align well with the dual goals of enhancing hydroponic growth and delivering long-term C benefits. These findings reinforce the importance of tailoring pyrolysis protocols to the characteristics of the feedstock, rather than applying a uniform temperature to different types of manure.

Also, specifications for quality control are necessary for the safe and effective use of digestate-derived biochar in soilless systems, where plant growth is very responsive to salinity and ionic composition of the substrate. The recommended limits for soilless systems, therefore, are: < 1.2–1.5 for EC (mS cm^−1^) (Joseph et al. 2021) (Table 2); and a pH within 5.5–6.5 is recommended for most species (Balliu et al. 2021).

**Table 2:** Electrical conductivity (EC) (mS cm⁻^1^) threshold values commonly recommended for soilless and hydroponic systems

EC threshold (mS cm⁻^1^)	Agronomic meaning	References
0.8 – 1.2	Ideal range for nutrient-film and floating-raft hydroponics; optimal nutrient uptake under low osmotic pressure	Balliu et al. (2021)
< 1.2 – 1.5	Recommended limit for soilless substrates to avoid salt stress and maintain stable nutrient availability	Joseph et al. (2021)
< 2.0	Maximum tolerated by most germinating seeds and young roots in hydroponic fodder systems	Gupta and Huang (2014)
> 2.5	Salt-induced osmotic inhibition begins to sharply reduce maize biomass and chlorophyll	Didaran et al. (2024)

Nevertheless, the influences of biochar on plant growth are context-specific, as each plant has different growth responses to biochar and different tolerances to certain kinds of stress. Also, it should be considered that biochar made from different raw materials mixed with components of substrates used for soilless cultivation will have variable effects that would need to be assessed and compared to any potential combination with the effects of the other component’s percentage or dose. According to the results obtained, and taking into account the considerations mentioned above, the BDST–500 treatment is the one chosen to evaluate its use as a substrate for hydroponic forage crops. The selection is based on its higher carbon content, which is related to a slower decomposition over time, making it durable as an inert medium (see Table 1). In addition, its lower volatile matter content makes it less phytotoxic, gives it greater stability as a substrate, lower microbial oxygen consumption, and less competition for nutrients.

The industrial scale-up potential of BDST–500 biochar should also be considered. While dairy slurry is readily available in intensive farming regions, the cost of pyrolysis at 500 °C may be a limiting factor due to energy inputs and infrastructure requirements (Rathnayake et al. 2023). However, emerging approaches such as medium-scale mobile pyrolysis units, which enable on-site biochar production from farm wastes, have been shown to reduce transport costs and increase adoption potential (John et al. 2024). Additionally, co-feeding pyrolysis reactors with other agricultural wastes (e.g., crop residues or green waste) could enhance throughput and improve the economic feasibility through cost sharing and by-product utilization (Gusiatin et al. 2024).

### Agronomic response of fodder to biochars

The agronomic response of hydroponic corn forage to the application of different biochars varied significantly depending on the type of digestate used as an amendment (Table 3). At day 0, after equilibration with biochar, solution pH and EC were slightly higher than the control and scaled with biochar feedstock salinity. BDST produced the smallest shifts (pH ≈ 6.2–6.3; EC ≈ 1.8–2.0 mS cm⁻^1^), BDSP intermediate (pH ≈ 6.3–6.6; EC ≈ 2.0–2.3 mS cm⁻^1^), and BDSV the highest (pH ≈ 6.6–6.9; EC ≈ 2.3–2.6 mS cm⁻^1^). After 8 days, moderate drifts were observed (ΔpH ≤  + 0.7; ΔEC ≤  + 0.5), remaining within agronomic ranges. These chemical shifts help to explain the observed differences in germination and seedling vigor, as higher solution EC in high-dose BDSP and BDSV treatments was associated with reduced performance. Table 3 presents the means and their respective standard deviations for the variables evaluated. The results indicate that the dose and type of digestate-derived biochar significantly influenced plant growth parameters, depending on both the digestate source (BDSP, BDST, BDSV) and the applied pyrolysis temperature (400, 500, and 600 °C). Overall, biochar produced from BDST at 500 °C promoted the highest chlorophyll (SPAD), fresh weight, and dry weight values, regardless of the dose. In particular, dose C4 (BDST–500 °C) was the most effective, reaching 12.2 SPAD chlorophyll, 34.9 g fresh weight, 12.7 g dry weight, and 79.6% moisture, indicating a highly positive effect on forage development. In contrast, BDSP biochar treatments, especially at 400 °C, presented the lowest chlorophyll and biomass values, suggesting a lower capacity to stimulate plant growth (Table 3). In summary, both the pyrolysis temperature and the type of digestate determine the agronomic properties of biochar and its effectiveness as an amendment in hydroponic systems.

**Table 3:** Mean values (± standard deviation) of physiological and productivity variables, chlorophyll content (SPAD), Fresh weight (g), Dry weight (g), and Moisture (%) in maize fodder grown under different biochar treatments

Biochar	T (°C)	Dose	Chlorophyll (SPAD)	Fresh weight (g)	Dry weight (g)	Moisture (%)
BDSP	400	C1	3.5 ± 0.2	21.5 ± 3.2	9.6 ± 1	62.1 ± 0.6
		C2	3.2 ± 0.8	21.1 ± 1.2	9.3 ± 0.4	62.2 ± 2.5
		C3	3.0 ± 1.9	21.0 ± 0.5	9.6 ± 0.6	60.6 ± 1.8
		C4	3.1 ± 1.3	18.5 ± 0.2	9.9 ± 0.3	60.7 ± 2.6
	500	C1	2.8 ± 0.4	21.9 ± 3.4	9.8 ± 0.1	58.8 ± 0.8
		C2	2.6 ± 1.1	22.5 ± 2.5	9.7 ± 0.3	60.6 ± 0.5
		C3	2.8 ± 0.4	20.3 ± 1.4	9.2 ± 0.1	58.5 ± 2.4
		C4	3.6 ± 1.2	22.4 ± 2.2	9.8 ± 0.7	62.9 ± 1.4
	600	C1	2.6 ± 1.2	21.8 ± 2.2	9.4 ± 0.5	61.4 ± 1.7
		C2	3.3 ± 2.3	23.0 ± 1.8	9.9 ± 0.2	62.1 ± 1.6
		C3	3.0 ± 1.5	21.1 ± 1.6	9.6 ± 0.4	60.7 ± 1.9
		C4	3.8 ± 1.2	19.5 ± 0.4	9.2 ± 0.5	61.7 ± 0.6
BDSV	400	C1	6.5 ± 0.5	26.2 ± 1.9	10.5 ± 0.2	68.1 ± 2.5
		C2	6.2 ± 1.1	25.1 ± 0.3	10.4 ± 0.2	68.1 ± 1.9
		C3	6.0 ± 0.7	25.2 ± 2.3	10.7 ± 0.4	67.6 ± 3.2
		C4	5.9 ± 0.7	26.9 ± 1.2	10.7 ± 0.4	67.8 ± 1.4
	500	C1	4.8 ± 0.3	23.7 ± 1.1	10.4 ± 0.4	68.9 ± 3.4
		C2	6.1 ± 0.4	25.0 ± 2.4	10.4 ± 0.8	66.1 ± 0.8
		C3	4.5 ± 0.4	24.6 ± 1.7	10.4 ± 0.7	66.9 ± 1.6
		C4	5.1 ± 0.8	23.8 ± 2.7	10.2 ± 0.5	66.4 ± 1.6
	600	C1	5.5 ± 0.5	24.5 ± 1.1	10.6 ± 0.4	68.6 ± 2.7
		C2	5.4 ± 1.6	24.1 ± 3.1	10.2 ± 0.3	68.7 ± 3.2
		C3	6.5 ± 1.3	24.5 ± 1.4	9.9 ± 0.2	68.9 ± 2.1
		C4	6.4 ± 0.8	23.9 ± 1.4	10.5 ± 0.5	67.6 ± 1.2
BDST	400	C1	10.0 ± 1.2	29.8 ± 1.0	11.2 ± 0.8	76.9 ± 3.5
		C2	9.8 ± 0.9	30.4 ± 0.9	11.8 ± 0.9	74.1 ± 2.6
		C3	9.5 ± 1.1	31.0 ± 1.6	11.2 ± 0.6	73.7 ± 1.9
		C4	10.0 ± 0.9	30.1 ± 1.2	11.0 ± 0.3	73.2 ± 3.1
	500	C1	9.4 ± 1.1	30.1 ± 1.4	11.6 ± 0.1	74.1 ± 2.2
		C2	9.0 ± 0.3	28.0 ± 2.4	11.5 ± 0.3	75.5 ± 1.7
		C3	9.4 ± 0.4	31.4 ± 2.7	11.4 ± 0.2	73.3 ± 2.6
		C4	12.2 ± 0.4	35.0 ± 1.6	12.7 ± 0.5	79.7 ± 2.2
	600	C1	9.6 ± 0.6	28.9 ± 3.0	11.4 ± 0.4	74.4 ± 1.7
		C2	9.4 ± 0.8	31.4 ± 2.0	11.9 ± 0.5	73.2 ± 2.5
		C3	8.3 ± 0.5	31.4 ± 1.9	11.4 ± 0.4	74.8 ± 1.3
		C4	8.3 ± 1.1	28.2 ± 1.2	10.9 ± 0.2	73.7 ± 0.5

Treatments varied by biochar type (BDSP, biochar from swine; BDSV, biochar from cattle; BDST, biochar from dairy); pyrolysis temperature (400, 500, and 600 °C), and biochar dose (C1: 0 g, C2: 1.25 g, C3: 2.5 g, C4: 6.25 g per container)

**Fig. 5:**
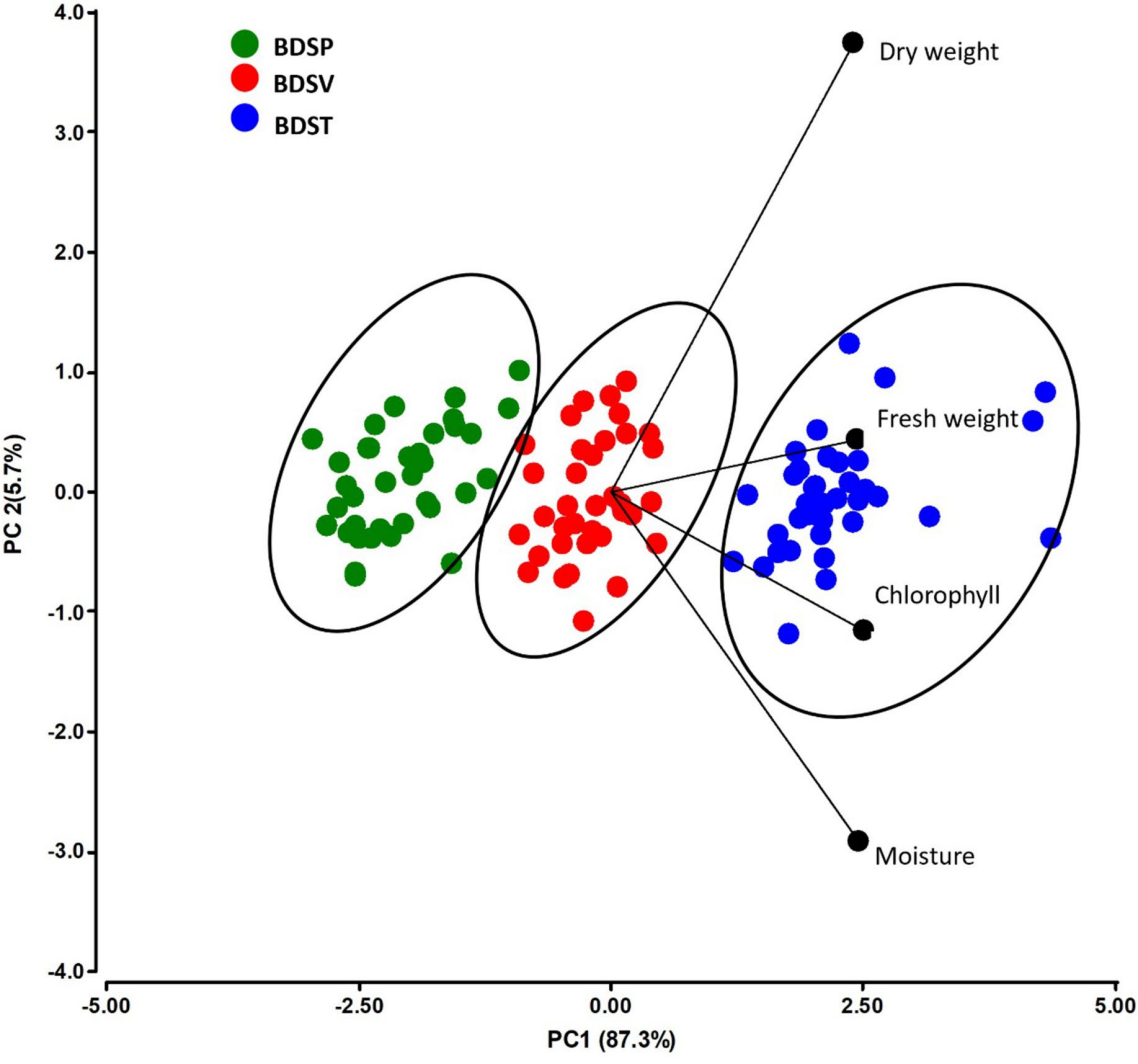
Principal Component Analysis (PCA) biplot illustrating the distribution of treatments based on dry weight, fresh weight, chlorophyll content, and tissue moisture. Treatments are grouped by biochar type: BDSP (from swine digestate, green), BDSV (from cattle digestate, red), and BDST (dairy digestate, blue). Vectors represent the contribution of each variable to the principal components

The PCA explained a large portion of the total variance in the dataset, with PC1 accounting for 87.3% and PC2 for 5.7%. Together, they captured 93.0% of the variability in plant performance traits (Fig. 5). PC1 was primarily defined by positive loadings from dry weight, fresh biomass, and chlorophyll content, indicating that these traits were the main drivers of variance across treatments. PC2, although explaining a smaller portion of the variance, was influenced mostly by moisture content, which displayed a distinct vector orientation. The PCA biplot separated the treatments according to the type of biochar used. BDST clustered on the positive axis of PC1 and was strongly associated with high levels of fresh biomass, chlorophyll content, and dry weight. This grouping reflects a superior response in the plant under BDST-amended conditions, likely due to a combination of enhanced nutrient availability, favorable pH buffering, and reduced salt stress. As shown in previous sections, the BDST-500 exhibited lower EC and higher OM than BDSV and BDSP. These factors are known to promote root function and nutrient uptake in hydroponic systems (Ephraim et al. 2024). The strong association of BDST–500 with high chlorophyll and biomass in the PCA biplot (Fig. 5) indicates that these traits dominate the positive axis of PC1. This component, therefore, primarily reflects growth performance. In contrast, PC2 contributes to moisture variability and differentiates treatments with similar biomass but varying tissue water content. Hence, moisture appears as a secondary dimension of agronomic response.

The high total carbon and low O/C ratio in BDST biochar may also contribute to greater nutrient retention and moderated nutrient release, facilitating a more stable root environment (Ephraim et al. 2024). BDSV, red dots, occupied an intermediate zone in the PCA space. Their partial alignment with fresh and dry biomass suggests moderate efficacy; however, their weaker association with chlorophyll content or tissue moisture implies limited physiological enhancement. This performance pattern is consistent with the higher ash content and elevated salinity of BDSV, which can induce osmotic stress, restrict chlorophyll synthesis, or interfere with micronutrient availability. These phenomena have been reported previously in saline or ash-rich char amendments (Alburquerque et al. 2014; Abzazou et al. 2018). Moreover, recent studies employing multivariate statistical tools have further clarified the mechanisms behind such performance trends. For instance, Faloye et al. (2024) conducted a two-year field experiment assessing the combined effects of biochar and inorganic fertilizers on maize performance. Their principal component analysis revealed that nitrogen uptake, dry biomass accumulation, and soil moisture retention were dominant contributors to the first two components, strongly shaping plant response. These findings highlight the critical role of biochar physicochemical properties, such as ash content and feedstock origin, in modulating physiological parameters through effects on water and nutrient availability. The limited alignment of BDSV with chlorophyll and moisture traits in our dataset is therefore consistent with the broader literature, indicating that elevated salinity or ash content in biochar can dampen beneficial plant responses, especially in moisture-sensitive systems (Faloye et al. 2024).

BDSP, green dots, were positioned on the negative side of PC1 and were strongly associated with poor agronomic outcomes, particularly low dry biomass. This underperformance may stem from a combination of high ash content, elevated EC (exceeding 1100 µS cm ^−1^), and lower structural condensation (as reflected by their higher O content and H/C ratios). These features are typically associated with unstable organic fractions and a greater risk of phytotoxicity or salt accumulation, which have been observed in porcine-based biochars in other studies (Tsai et al. 2012; Iqbal et al. 2015). Although DSP biochars can provide nutrients, an imbalance between salt load and bioavailability may limit their effectiveness in hydroponic systems. To understand the mechanism of interaction between biochar source, pyrolysis temperature, and dose, it is important to consider the functional synergies among chemical and physical properties. For instance, dairy-based biochars at 500 °C combine low EC with high cation exchange capacity and moderate surface polarity. These properties together create an optimal root environment, reducing salt stress while promoting nutrient adsorption. The PCA also highlighted the strong alignment between dry weight and PC1, suggesting that this trait was the most influential in discriminating treatment performance. This interaction is agronomically meaningful: biochars with low EC and moderate aromaticity provide both stability and buffering capacity in the root zone, especially in non-soil systems. The dose threshold of 6.25 g per container emerges as a functional balance point, where nutrient availability is maximized without inducing water retention stress or ionic toxicity. In contrast, moisture showed a distinct vector orientation, indicating that it was less correlated with biomass-related outcomes and possibly acted as a limiting factor in some treatments.

The PCA also revealed that dry weight was the variable most closely aligned with PC1, indicating that it was the most influential trait of treatment performance. This result is consistent with previous research in hydroponics, in which dry biomass has been used as a reliable integrative indicator of nutrient efficiency and photosynthetic productivity (Shit et al. 2019; Khan et al. 2020). Moreover, comparative analysis with meta-analytic data confirms the superiority of BDST. Biederman and Harpole (2013) observed average yield gains of 28% from manure–based biochars below 1200 µS cm^−1^, similar to our EC range. Further, our results align with the work of Steiner et al. (2007), who emphasized the need to combine feedstock type, temperature, and application rate for optimal outcomes in terra preta systems. In contrast, moisture content exhibited an independent vector from biomass variables, suggesting that it contributed less to treatment discrimination and may have acted as a limiting or confounding factor. High tissue moisture does not always correlate with better physiological outcomes, especially under salinity stress conditions, where water may be retained passively rather than being used functionally for growth (Gupta and Huang 2014).

Overall, the multivariate distribution of treatments in PCA space clearly shows a hierarchy of biochar effectiveness: BDST > BDSV > BDSP. These differences confirm that the interaction between feedstock origin and pyrolysis conditions determines not only the chemical properties of biochar but also its biological compatibility with soilless systems. The strong association between BDST and improved plant performance reinforces the notion that biochar should be selected and optimized not only for its carbon stability or nutrient load, but also for how it interacts with plant physiology, especially under low buffering conditions such as those found in hydroponics.

The multivariate analysis of variance (MANOVA, Tables 4 and 5) revealed that the type of biodigestate used as feedstock for biochar production had a highly significant effect (partial η^2^ = 0.52, 95% CI [0.41–1.00], *p* < 0.001) on all evaluated physiological variables in forage: fresh weight (MANOVA, F = 214.2, *p* < 0.001), dry weight (MANOVA, F = 140.0, *p* < 0.001), chlorophyll content (MANOVA, F = 376.4, *p* < 0.001), and moisture (MANOVA, F = 376.4, *p* < 0.001). Model fit and predictive accuracy were evaluated through parity plots comparing observed and predicted values for each agronomic variable (Fig. S2, Supplementary Information). The strong 1:1 alignment across variables confirms that the factorial MANOVA adequately captured treatment effects and experimental variability. To further quantify these effects, univariate effect sizes were calculated for each variable (Table 6). Digestate type exhibited the largest impact, with high effect sizes observed for fresh weight (η^2^_p_ = 0.86 [0.81 – 1.00]), dry weight (η^2^_p_ = 0.80 [0.73 – 1.00]), moisture (η^2^_p_ = 0.91 [0.88 – 1.00]), and chlorophyll content (η^2^_p_ = 0.91 [0.88 – 1.00]). This confirms that the substrate origin is the primary determinant of forage physiological response, surpassing the influence of pyrolysis temperature and biochar dose, which showed minimal effect sizes (0.007–0.07, 95% CI spanning 0–1.00). These physiological traits are critical indicators of plant performance and nutritional quality. Chlorophyll, as the primary photosynthetic pigment, is directly linked to the photosynthetic capacity and metabolic health of plants (Simkin et al. 2022). In forage crops, higher chlorophyll content is also associated with improved nutrient density and feed value (Capstaff and Miller 2018). The higher chlorophyll content observed under BDST–500 treatments support the idea that dairy-derived biochar may enhance nitrogen assimilation or preserve microenvironmental conditions conducive to chlorophyll biosynthesis. This aligns with the findings of Khan et al. (2020), who reported improved leaf chlorophyll and plant growth when using manure- and nitrogen-enriched biochar. Under mild salinity, plants often accumulate Na^+^ and Cl^−^ ions that destabilize chlorophyll–protein complexes, leading to photoinhibition (Didaran et al. 2024). The markedly lower EC of BDST–derived chars therefore not only prevent osmotic stress but may safeguard the photosystems directly, explaining the ∼threefold higher SPAD values relative to BDSP. The positive correlation between SPAD and crude protein observed here (r = 0.81; *p* < 0.01) supports the use of chlorophyll as a rapid indicator of forage quality (Jagdeep-Singh and Varinderpal-Singh 2022; Shehab et al. 2023).

**Table 4:** Statistical results of the factorial MANOVA on Fresh weight (g), Dry weight (g), Chlorophyll (SPAD), and Moisture (%) variables in maize fodder bioassay and its interactions

Source of Variation	Fresh weight (g)		Dry weight (g)		Chlorophyll (SPAD)		Moisture (%)	
	F	*P*-Value	F	*P*-Value	F	*P*-Value	F	*P*-Value
Digestate type	214.2	***	140.03	***	376.4	***	376.4	***
Pyrolysis temperature	0.75	NS	1.07	NS	1.54	NS	0.76	NS
Biochar doses	0.93	NS	0.64	NS	0.16	NS	0.32	NS
Digestate type x Pyrolysis temperature^(a)^	2.19	***	1.92	***	3.03	***	2.09	***
Digestate type x Biochar doses^(b)^	1.86	***	0.43	***	0.48	***	0.95	***
Pyrolysis temperature x biochar doses^(c)^	2.24	***	1.49	***	1.20	***	2.22	***
Digestate type x Pyrolysis temperature x Biochar doses^(d)^	1.60	***	2.20	***	1.73	***	1.18	***

(a) digestate type × pyrolysis temperature, (b) digestate type × biochar dose, (c) pyrolysis temperature × biochar dose, and (d) digestate type × pyrolysis temperature × biochar dose. F and *p*-values are dimensionless

^***^ *p* < 0.001, NS indicates *p* > 0.05

**Table 5:** Multivariate effect sizes (MANOVA)

Effect	Partial η^2^	95% CI
Digestate type	0.52	[0.41–1.00]
Pyrolysis temperature	0.05	[0.00–1.00]
Biochar doses	0.05	[0.00–1.00]
Digestate type × Pyrolysis temperature	0.10	[0.01–1.00]
Digestate type × Biochar doses	0.07	[0.00–1.00]
Pyrolysis temperature × biochar doses	0.11	[0.00–1.00]
Digestate type × Pyrolysis temperature × Biochar doses	0.20	[0.01–1.00]

**Table 6:** Univariate effect sizes (ANOVA; partial η^2^ with 95% CI) for Fresh weight (g), Dry weight (g), Moisture (%), and Chlorophyll (SPAD)

Effect	Fresh weight (g)	Dry weight (g)	Moisture (%)	Chlorophyll (SPAD)
Digestate type	0.86 [0.81–1.00]	0.80 [0.73–1.00]	0.91 [0.88–1.00]	0.91 [0.88–1.00]
Pyrolysis temperature	0.02 [0.00–1.00]	0.03 [0.00–1.00]	0.007 [0.00–1.00]	0.04 [0.00–1.00]
Biochar doses	0.006 [0.00–1.00]	0.02 [0.00–1.00]	0.05 [0.00–1.00]	0.07 [0.00–1.00]
Digestate type x Pyrolysis temperature	0.11 [0.00–1.00]	0.10 [0.00–1.00]	0.10 [0.00–1.00]	0.14 [0.01–1.00]
Digestate type x Biochar doses	0.13 [0.00–1.00]	0.04 [0.00–1.00]	0.07 [0.00–1.00]	0.04 [0.00–1.00]
Pyrolysis temperature x biochar doses	0.16 [0.00–1.00]	0.11 [0.00–1.00]	0.16 [0.00–1.00]	0.09 [0.00–1.00]
Digestate type x Pyrolysis temperature x Biochar doses	0.21 [0.00–1.00]	0.27 [0.03–1.00]	0.16 [0.00–1.00]	0.22 [0.00–1.00]

Regarding dry matter content, treatments with higher values, such as BDST–500–C2, are of agronomic relevance. Dry matter content is directly related to forage quality, energy density, and storage stability (Bayhan 2023). Higher biomass values also correspond to enhanced nutrient-use efficiency and improved carbon allocation, supporting greater livestock productivity at lower feeding costs (Bayhan 2023). Conversely, high moisture values (e.g., > 63% in several BDSP treatments) may suggest excessive water retention without corresponding tissue development, a phenomenon often observed in environments prone to stress or substrates with poor aeration. Additionally, elevated moisture levels can negatively affect forage conservation and increase the risk of spoilage, whereas excessively low moisture can reduce palatability and digestibility (Wilkinson et al. 2018; Katoch 2022). The observed variability in forage moisture depending on digestate type and pyrolysis temperature suggests the importance of considering these factors during production and post-harvest management. Organic amendments, such as biochar, can influence these parameters by enhancing nutrient availability and modifying soil structure (Shyam et al. 2025). Notably, the interaction between pyrolysis temperature and digestate type affects dry biomass response, indicating that thermal optimization may be key to maximizing forage productivity.

These findings underscore that the initial substrate composition (DSP: swine, DSV: cattle, DST: dairy) is a key factor in the physiological response of plants, likely due to differences in nutrient ratios, OM, microbial load, and trace elements retained after pyrolysis. Comparable effects of feedstock have been reported in soil systems, where manure-based biochar differs markedly in terms of N, P, and salinity, resulting in contrasting plant responses (Laird et al. 2010; Manolikaki and Diamadopoulos 2019).

Among the three digestate types, BDST elicited the most favorable physiological responses. The treatment BDST–500–C4 (6.5 g per container) exhibited the highest mean chlorophyll content (6.1 SPAD units), as well as one of the highest fresh weight values (30.2 g per container) and dry weight values (13.1 g per container), along with relatively moderate moisture content (56.6%). This indicates a favorable balance between biomass accumulation and functional water use. This trend can be partially attributed to its higher total carbon content (up to 60.4% at 500 °C) and OM (up to 71.6% at 400 °C), suggesting greater availability of functional compounds and bioactive structures that may enhance plant growth. DST presented the lowest EC values among all treatments (746–916 µS cm^−1^) among all treatments, indicating a lower concentration of soluble salts and thus a reduced risk of osmotic stress in plants. Similar low EC biochars have been shown to promote root elongation and nutrient acquisition in hydroponic tomato and barley trials (Zhang et al. 2020; Luigi et al. 2022). The elevated dissolved OC and moderate pH buffering of BDST created a more hospitable rhizosphere, facilitating chlorophyll synthesis and biomass accumulation. In contrast, BDSP obtained at 500 °C (DSP–500–C3) showed significantly lower chlorophyll content (1.7 SPAD), with reduced fresh and dry weights. This confirms the poorer agronomic performance of pig slurry-derived amendments under these conditions.

The BDSP and BDSV digestates had higher ash contents (up to 35.5% and 33.6%, respectively), suggesting that they contain a greater proportion of non-volatile mineral compounds. While these compounds may contribute nutrients, their presence in excess can reduce water retention capacity or negatively affect root development. Notably, BDSV showed the highest EC values (up to 2360 µS cm^−1^ at 500 °C), which could potentially induce salt stress and limit water retention, a pattern also observed by Alburquerque et al. (2014) for ash-rich olive mill waste biochar, which lowered field capacity and impaired sunflower growth.

Conversely, pyrolysis at temperatures of 400, 500, and 600 °C alone did not produce significant effects on any of the physiological variables (Tables 4 and 5). This suggests that, within the evaluated range, the physicochemical transformations induced by pyrolysis were insufficient to substantially alter nutrient availability or water-holding capacity in ways that affect forage physiology. This result aligns with Joseph et al. (2021), who emphasized that plant responses often emerge from complex synergies among biochar chemistry, application environment, and plant species rather than from temperature per se. Similarly, Ashworth et al. (2014) observed that switchgrass yield was more affected by feedstock identity than by pyrolysis temperature between 400 and 550 °C.

Similarly, the dose of biochar applied (0, 1.25, 2.50, and 6.25 g per container) had no significant main effects on fresh weight, dry weight, chlorophyll content, or moisture, indicating that the quantity applied was not the primary determinant of biological response under these conditions (Tables 3 and 5). An increasing amount of work points to there are threshold or saturation patterns: once a minimal amount of biochar supplies sufficient sorption sites and buffering capacity, further additions yield diminishing returns or may even impede root aeration (Malik et al. 2022). Here, significant interactions reveal that the efficacy of the dose is conditional on feedstock chemistry and pyrolysis history.

Significant interactions were observed between digestate type and pyrolysis temperature, digestate type and biochar dose, temperature and dose, as well as in the three-way interaction, all showing highly significant values (*p* < 0.001) (Table 4). Notably, the interactions between factors revealed moderate effect sizes, emphasizing that the response of plants to biochar is context-dependent (Table 6). These results indicate that the effects of biochar on plant growth are not determined by individual factors alone, but rather by the specific combination of digestate type, pyrolysis temperature, and applied dose (η^2^_p_ = 0.20 [0.01–1.00], Table 5). In particular, the interaction between digestate type and pyrolysis temperature was significant for all evaluated variables (MANOVA, *p* < 0.001), highlighting that plant response depends not only on the feedstock characteristics but also on the transformations induced by thermal treatment. For instance, while a given temperature may enhance the agronomic properties of BDST, it may be less effective or even detrimental when applied to BDSP or BDSV. This is supported by the FC content, which increased notably with temperature in BDSV (46.8%) and BDST (50.9%), whereas the improvement was less pronounced in BDSP (Table 1).

Additionally, oxygen content decreased with higher temperatures in all cases, reflecting greater carbon stability, though not necessarily result in enhanced plant growth. Even though higher temperatures increase FC and reduce H/O ratios, the agronomic outcome depends on the original feedstock. For instance, improved H/C and O/C ratios may only enhance water retention when the feedstock is favorable, porosity and functionality vary across digestates, as observed by Wang et al. (2019). Likewise, the interaction between digestate type and biochar dose was significant (MANOVA, *p* < 0.001), suggesting that the response to the quantity applied is modulated by the biochar’s chemical characteristics. Biochars that are richer in specific compounds (e.g., labile O/C or nitrogen) might be more effective at low doses, whereas others require higher doses to have an effect. This may be linked to differences in the C/N ratio, the presence of phytotoxic compounds, or water retention properties.

The interaction between pyrolysis temperature and dose was also statistically significant (MANOVA, *p* < 0.001), indicating that the agronomic performance of biochar is not determined solely by dosage, but also by its thermal history (Table 4). Pyrolysis-induced changes in porosity, surface area, and cation exchange capacity may alter the system’s response to different levels of biochar. Higher temperatures generate larger, more hydrophobic pores that can enhance aeration at moderate doses, but create drought-like microsites when overloaded, mirroring the non-linear trends observed by Malik et al. (2022) in lettuce grown under biochar of different particle sizes. These interactive effects reinforce the importance of assessing dosage in the context of specific biochar properties, as highlighted by the aforementioned studies.

Finally, the three-way interaction between digestate type, pyrolysis temperature, and applied dose was significant for all variables (MANOVA, *p* < 0.001), demonstrating the complex nature of the biochar effects and the need for multivariable analysis to select the most effective agricultural applications (Table 4). This confirms that the efficacy of biochar as a soil amendment cannot be evaluated in isolation, but rather through the synergy among the three interacting factors. This finding is consistent with Steiner et al. (2007), who demonstrated that optimized combinations of feedstock, temperature, and dose in Terra Preta soils dramatically improved productivity and nutrient retention. Recent greenhouse trials with nitrogen-enriched biochars have reported increases of up to 28% in chlorophyll and 47% in plant height (Khan et al. 2020), corroborating our finding that the nutrient-dense DST combination delivers superior physiological performance. Plant responses to biochar are governed by the interplay of digestate chemistry, pyrolysis conditions, and application rate rather than by any single factor. Dairy digestate consistently produced the most favorable outcomes, warranting further investigation into its high humic-acid fraction, balanced macro-/micronutrient profile, and moderate salinity. Continuous refinement of these interacting variables should guide the design of bespoke biochars tailored to hydroponic forage systems.

Recent meta‑analyses corroborate the findings observed in this work. For instance, Biederman and Harpole (2013) reviewed 109 experiments and reported that biochars produced from animal manures increased above‑ground biomass by 28% on average, provided the EC remained below 1200 µS cm^−1^, precisely the salinity window in which BDST‑500 operates. Likewise, Hu et al. (2023) showed that co‑application of dairy slurry biochar and compost improved maize yield by 2.5 times compared to the control, linking the response to greater cation exchange capacity and enhanced N retention, mechanisms that are also inferred from the higher protein content observed in the BDST treatments.

During pyrolysis, part of the organic nitrogen from the feedstock is retained in thermally stable aromatic structures (e.g., pyridinic or pyrrolic N), which act as slow-release sources of plant-available nitrogen (Joseph et al. 2021). Manure-derived biochars also enhance the cation exchange capacity and stimulate microbial mineralization processes, improving nitrogen retention and augmented efficiency. Increased N availability is associated with higher chlorophyll synthesis and, consequently, greater crude protein accumulation in plant tissues, which is illustrated by a positive correlation between SPAD and crude protein (Yuan et al. 2022).

Several studies have shown that biochar’s effects are strongly modulated by feedstock chemistry and pyrolysis conditions. For instance, Krounbi et al. (2021) reported that manure-based biochars have higher mineral and salt content compared to woody feedstocks, which can affect nutrient dynamics in soilless systems. Similarly, Botyanszká et al. (2024) observed that interactions between biochar dose and particle size impact root oxygenation and water availability, which are critical in hydroponic settings.

In this study, the superior performance of BDST–500 likely emerges from a synergy between its chemical composition, moderate ASH, low EC, high C, and OM, and the optimal thermal structuring achieved at 500 °C, which maximizes its adsorptive surface while maintaining functionality. This combination favors nutrient buffering without excessive salinization and may stimulate root microbe symbioses, a hypothesis supported by reports of increased microbiota activity in biochar-amended substrates (Kracmarova et al. 2024). In practical terms, understanding these interactions allows for the customization of biochars based on both agronomic targets and processing logistics, thereby enhancing field-level applicability and circular bioeconomy outcomes.

Limitations of the present study must also be acknowledged. While the 8-day controlled laboratory experiment provided a rapid and cost-efficient method to screen the agronomic effects of different biochar treatments, it represents an early-stage approach that may not fully capture long-term field performance. Short-cycle systems such as the one used here are relevant for fast-growing crops like hydroponic forage, where plants are harvested within days. However, this limited duration restricts the observation of biochar’s cumulative effects on root-microbe interactions, nutrient release kinetics, or structural changes in the substrate over time. Consequently, future experiments should explore extended cultivation periods, multiple harvest cycles, and environmentally variable scenarios to validate the robustness and scalability of these findings. Despite these limitations, the short-term design remains valuable for identifying promising biochar feedstock temperature combinations that warrant further development and field validation.

### Effect of biochar on crude protein content in soilless fodder systems

**Fig. 6:**
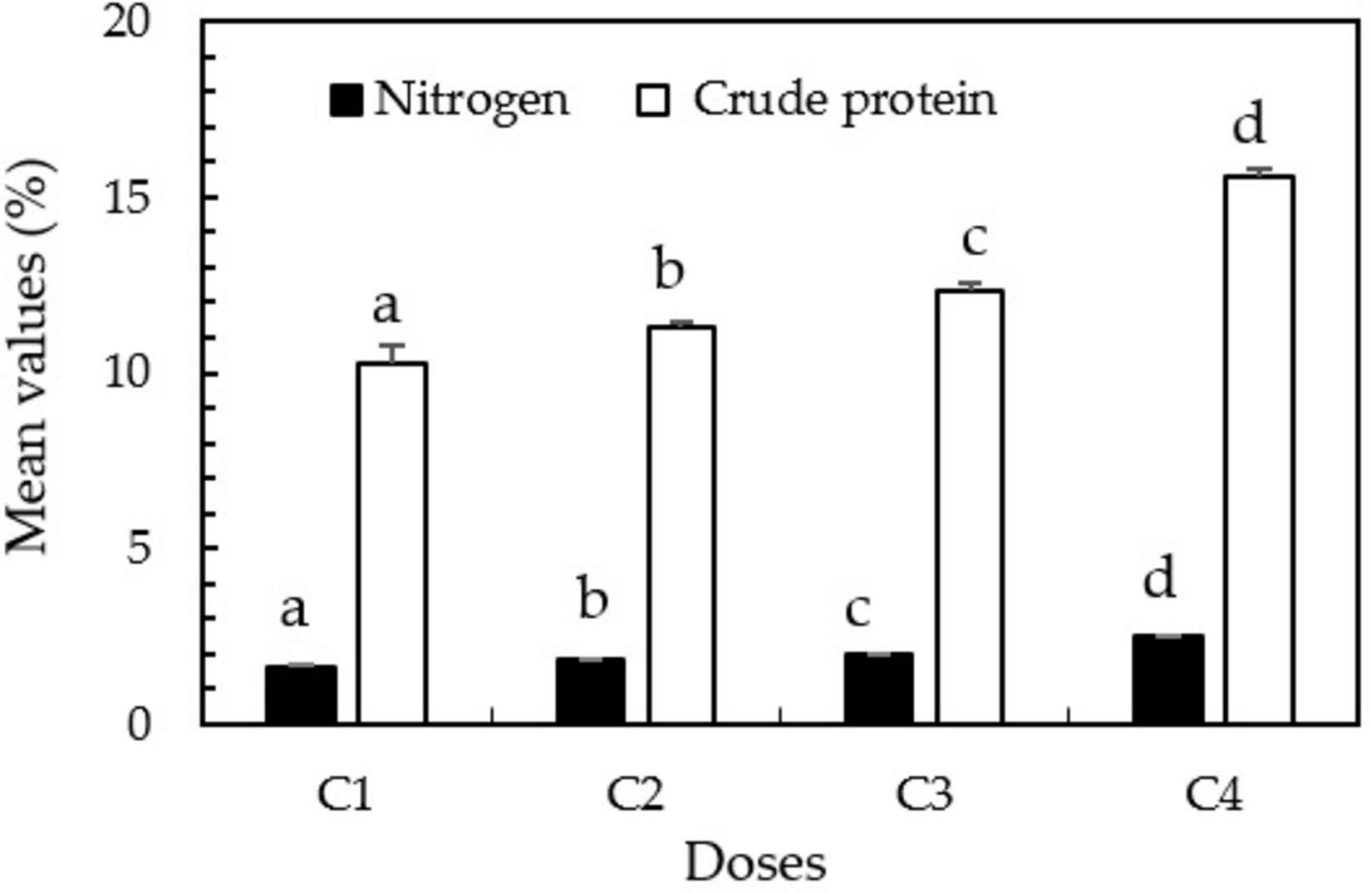
Mean values (± standard deviation) of nitrogen and crude protein content in maize fodder across increasing doses of BDST–500 (biochar from swine digestate obtained at 500 °C) (C1: 0 g, C2: 1.25 g, C3: 2.5 g, C4: 6.25 g per container). Different superscript letters indicate statistically significant differences between treatments according to Tukey’s HSD test (*p* < 0.05)

Following the evaluation of the physicochemical characteristics of the different biochars and their impact on forage performance (fresh weight, dry weight, moisture content, and chlorophyll levels), the BDST–500 was identified as the best-performing treatment overall. Therefore, its nutritional contribution was assessed further by analyzing total nitrogen and crude protein content in the forage at four application rates (C1 to C4). As shown in Fig. 6, increasing doses of BDST–500 biochar led to a progressive and statistically significant increase in both nitrogen and crude protein content in maize fodder. Nitrogen concentration rose from 1.6 ± 0.1% in C1 to 2.5 ± 0.03% in C4, while crude protein content increased from 10.3 ± 0.5% to 15.6 ± 0.2%. Each dose level showed significant differences from the others, as indicated by distinct superscript letters (Tukey’s HSD, *p* < 0.05). These results suggest that higher doses of BDST–500 biochar enhance the nutritional quality of hydroponic maize fodder by improving its nitrogen assimilation and protein accumulation. These effect sizes are agronomically relevant, as an increment of 3–4 percentage units in crude protein can translate into 0.15–0.20 kg day^−1^ additional milk yield in dairy cattle diets (Magan et al. 2021).

To further characterize the dose–response relationship, the association between BDST–500 biochar dosage and crude protein content was modeled statistically. The relationship was well fitted to a linear model (*p* < 0.001), with no evidence of curvature (*p* = 0.79 for the quadratic term). The linear model explained 98% of the observed variability (R^2^ = 0.98), indicating a monotonically increasing response within the evaluated range (0–6.25 g). A nonlinear Michaelis–Menten model did not improve the fit (ΔAIC =  + 70), confirming the absence of saturation within the tested doses (Fig. S3, Supplementary Information). The effective upper limit of the range, therefore, corresponds to the maximum dose tested (6.25 g), which yielded the highest crude-protein values without signs of plateauing.

The significant increase in protein levels at 6.25 g per container (C4) indicates that, under the EC window provided by DST biochar (≈ 916 µS cm^−1^), a high dose can enhance nitrogen assimilation without causing the salt stress commonly reported for manure-based chars (Kamal et al. 2024). Two complementary mechanisms may explain this response. First, greater availability of labile-N: dairy-slurry biochars retain ammonium and organic N on functionalized surfaces and gradually release them, promoting a sustained nitrogen supply to roots (Krounbi et al. 2021). Second, enhanced cation-exchange capacity (CEC) and microbial activity increase the biochar fraction, amplifying microsites for nitrifying and diazotrophic microbes, as demonstrated by Seyedghasemi et al. (2021) for manure chars in soilless systems. From a nutritional standpoint, increasing crude protein from 10 to 15.6% reduces the need for external protein concentrate by ≈ 20% in dairy rations targeting 16% CP (Magan et al. 2021). This translates into tangible savings in feed cost and closes nutrient loops by recycling dairy effluents into high-quality fodder, an outcome aligned with circular-bioeconomy goals (Harrison et al. 2022). Finally, the positive, near-linear association between SPAD chlorophyll readings and crude protein content (r = 0.81, *p* < 0.01) corroborates the use of chlorophyll as a rapid proxy for forage nutritive value (Peters et al. 2022). Together, these findings confirm that BDST–500 °C delivers maximal agronomic and nutritional benefits at the highest tested dose (C4), without surpassing critical salinity thresholds, and should therefore be prioritized for further on-farm validation.

Beyond its demonstrated efficacy in hydroponic systems, BDST biochar also shows strong potential for broader agricultural applications, particularly in soilless pot cultivation and vertical farming systems. The favorable physicochemical traits of BDST, such as its balanced EC, enhanced capacity exchange, and organic matter content, could benefit root development and nutrient retention across a variety of soilless substrates. Moreover, its proven ability to enhance nitrogen assimilation and protein accumulation suggests its applicability in high-density production models where nutrient use efficiency is critical. Future trials should explore its compatibility with different growth media and irrigation regimes to validate its versatility under commercial and controlled-environment conditions.

### Techno-economic considerations and scalability

The laboratory-scale drying and pyrolysis methods used in this study prioritize experimental reproducibility and are not directly economically viable at scale. However, they provide the critical data needed to assess the potential for an integrated, circular system. In a commercial scenario, energy-intensive oven-drying would be replaced by efficient alternatives such as solar drying or waste-heat recovery from the pyrolysis process itself (Meyer et al. 2011; Capossio et al. 2022).

To frame this potential, a preliminary energy balance is instructive. The industrial scale-up potential of BDST–500 biochar should also be considered from an energy balance and economic perspective. A back-of-the-envelope estimate provides a preliminary feasibility assessment. The energy required for slow pyrolysis is highly variable but is estimated to be in the range of 2–5 MJ kg^−1^ of dry feedstock for a well-insulated, continuous system, with higher temperatures demanding more energy (Roberts et al. 2010). Assuming a conservative 4 MJ kg^−1^ to process dairy digestate at 500 °C and a typical biochar yield of 50% from digestate, the energy cost per kg of BDST–500 biochar produced is approximately 8 MJ kg^−1^.

Contrasting this input with the agronomic output from our study reveals the potential value. The optimal treatment (BDST–500 at 6.25 g per container) yielded a 1.1 g increase in dry fodder mass per container over the control. This translates to a dry mass yield gain of approximately 176 g kg^−1^ of applied biochar (1.1 g gain 0.00625 kg biochar^−1^).

From a livestock feed perspective, this gain must be valued against the cost of production. While the energy input for pyrolysis is non-trivial, it must be weighed against the concurrent benefits of waste management, the potential for energy recovery from syngas co-produced during pyrolysis to offset operational costs, and the production of a higher-value, protein-enriched fodder that can reduce reliance on external feed concentrates (Magan et al. 2021). Future techno-economic analyses incorporating capital costs, logistics, and valorization of all process streams are essential to confirm the commercial viability of integrating on-farm pyrolysis into hydroponic fodder systems.

## Conclusions

This study demonstrates that the origin of anaerobic digestate used as feedstock for biochar production substantially influences both the physicochemical characteristics of the resulting biochar and its agronomic performance in hydroponic forage systems. Among the three feedstocks assessed, dairy-derived biochar (BDST), particularly when produced at 500 °C and applied at a rate of 6.25 g per container, consistently delivered the most favorable outcomes in terms of dry biomass accumulation, chlorophyll content, and moisture retention, without contributing to salinity stress.

While pyrolysis temperature affected key biochar parameters such as ash content, EC, and fixed carbon, it had a limited independent impact on plant physiology. Instead, significant interactions among digestate type, pyrolysis temperature, and application dose were observed, highlighting the complexity of biochar plant dynamics and the necessity for multivariable approaches in agronomic evaluations. The application alone was not a primary determinant of performance but exhibited synergistic effects when combined with specific thermal and feedstock conditions, revealing response thresholds relevant to nutrient uptake and physiological efficiency.

A key finding is that BDST–500 at 6.25 g enabled optimal nitrogen assimilation and protein synthesis, likely due to its improved cation exchange capacity and structural properties that support microbial colonization and root development (Kracmarova-Farren et al. 2024). These results point to the potential of BDST biochar as a precision amendment in sustainable, resource-efficient agriculture. However, the study is limited by its short experimental duration (8 days) and the controlled hydroponic setup, which may not fully replicate soil or field conditions.

Although the present experiment was conducted over a short 8-day cultivation window and under a static soilless system without nutrient recirculation or dynamic nutrient replacement, the outcomes provide a robust basis for understanding nutrient efficiency and fodder quality under controlled conditions. Future studies extending the cultivation period and testing under dynamic hydroponic systems are warranted to confirm the broader applicability of these findings.

While this full factorial design successfully identified the most influential factors and their interactions, future research should employ advanced Design of Experiments (DoE) methodologies, such as Response Surface Methodology (RSM), for precise optimization. A focused study on the dairy-derived biochar (BDST), using a Central Composite Design with pyrolysis temperature and application dose as continuous factors, would be highly valuable. This approach would enable the generation of predictive models to locate the exact optimum combination that maximizes fodder yield and nutritional quality while explicitly constraining EC to non-phytotoxic levels, thereby defining clear operational windows for commercial application.

Moreover, future research should explore the long-term effects of BDST biochar under diverse environmental scenarios, including pot trials, vertical farming, and conventional soil-based agriculture, to validate its broader applicability and optimize formulation strategies. In addition, incorporating molecular and microbiome level analyses could help elucidate the mechanisms underlying biochar plant interactions and microbial modulation within the rhizosphere, which were beyond the scope of the present study but represent a valuable direction for future research. Overall, this work contributes to the growing evidence that biochar properties must be matched with intended agronomic contexts, reinforcing the role of tailored biochar systems within circular bioeconomy frameworks.

## Supplementary Information

Below is the link to the electronic supplementary material.

Supplementary Material 1

## Data Availability

The datasets used and/or analysed during the current study are available from the corresponding author on reasonable request.

## Abbreviations

BDSP: Biochar from swine digestate
BDST: Biochar from dairy digestate
BDSV: Biochar from cattle digestate
C: Carbon content (%)
Ca: Calcium
DSP: Swine digestate
DST: Dairy digestate
DSV: Cattle digestate
EC: Electrical conductivity (μS cm^−^^1^)
FC: Fixed carbon content (%)
H: Hydrogen content (%)
H/C: Hydrogen-Carbon molar ratio, dimensionless
K: Potassium
Mg: Magnesium
O: Oxygen content (%)
O/C: Oxygen–Carbon molar ratio, dimensionless
OC: Organic carbon (%)
OM: Organic matter (%)
P: Phosphorus
PCA: Principal Component Analysis
VM: Volatile matter (%)
