# Supplementary material for: Conversion of anaerobic digestates into biochar for sustainable fodder production in soilless agriculture
Source: Bioresour Bioprocess. 2026 Apr 2;13(1):49. doi: 10.1186/s40643-026-01014-7 (PMC13047021; doi:10.1186/s40643-026-01014-7)
Supplement: Supplementary file 1 — Supplementary Material 1 [file 40643_2026_1014_MOESM1_ESM.docx]

**Table S1:** Multivariate normality test using Mardia's skewness and kurtosis test and homogeneity of variance-covariance matrices using Box's M test.

|  | **Test** | **Variable** | **Statistic** | **gl** | **p_value** |
| --- | --- | --- | --- | --- | --- |
| Multivariate normality | Skewness |  | 11.145 | - | 0.942 |
|  | Kurtosis |  | -1.778 | - | 0.075 |
| Homogeneity of variance-covariance matrices | Box's M-test | Digestate type | χ² = 23.245 | 20 | 0.276 |
|  |  | Pyrolysis temperature | χ² = 14.052 | 20 | 0.827 |
|  |  | Biochar doses | χ ² = 20.977 | 30 | 0.888 |
|  |  | Digestate type x Pyrolysis temperature | χ² = 79.123 | 80 | 0.506 |
|  |  | Digestate type x Biochar doses | χ² = 100.41 | 110 | 0.732 |
|  |  | Pyrolysis temperature x biochar doses | χ² = 77.006 | 110 | 0.992 |

**Table S2:** Verification of the univariate assumptions of the MANOVA.

| **Dependent variable** | **Normality test (Shapiro–Wilk)** |  | **Homogeneity of variances test (Levene)** |  |
| --- | --- | --- | --- | --- |
|  | **W** | **p-valor** | **F** | **p_value** |
| Fresh weight | 0.983 | 0.191 | 0.366 | 0.999 |
| Dry weight | 0.995 | 0.967 | 0.525 | 0.980 |
| Chlorophyll | 0.991 | 0.730 | 0.548 | 0.973 |
| Moisture | 0.978 | 0.076 | 0.548 | 0.973 |


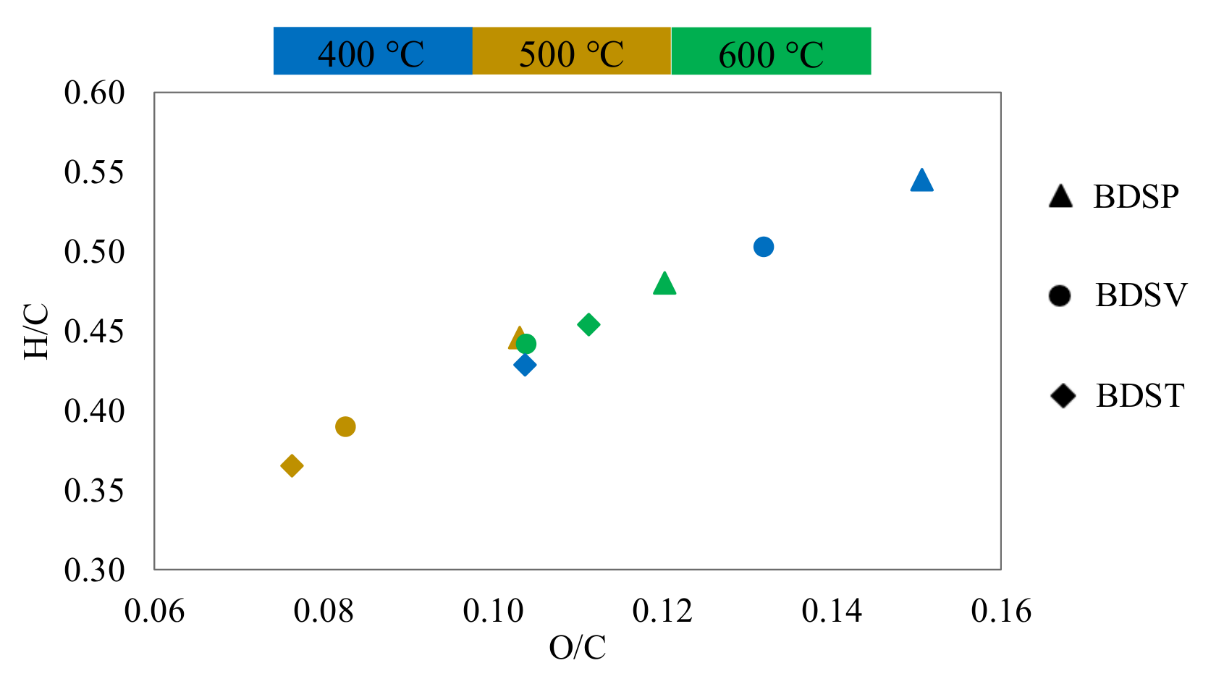


**Supplementary Fig. S1**. H/C and O/C molar ratios of BDSP (swine digestate), BDSV (cattle digestate), and BDST (dairy digestate) obtained at 400 °C, 500 °C, and 600 °C.


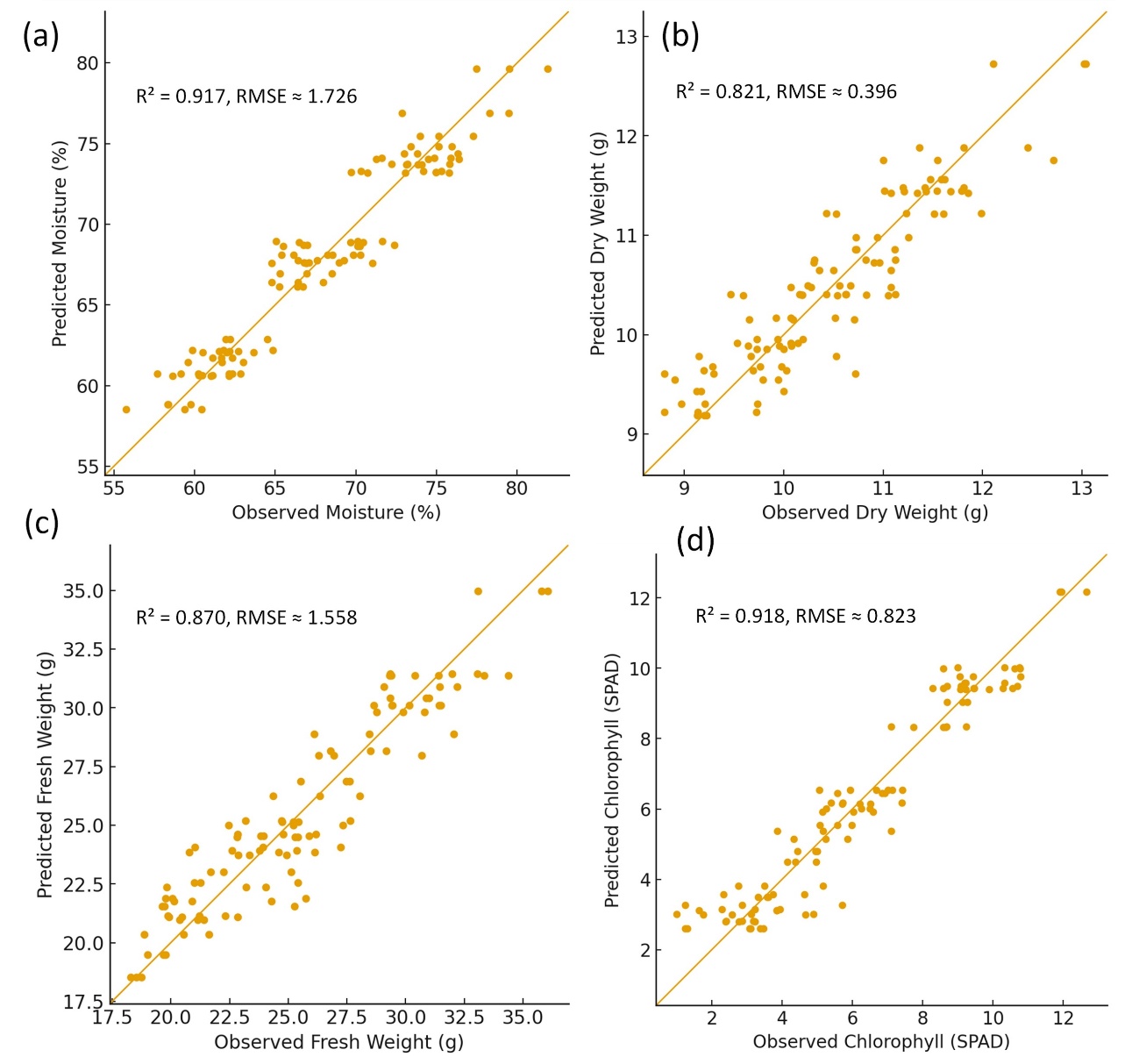


**Fig. S2.** Parity plots showing the relationship between predicted and observed agronomic variables obtained from the factorial MANOVA model: (a) moisture (%), (b) dry weight (g), (c) fresh weight (g), and (d) chlorophyll (SPAD). The 1:1 line indicates perfect model agreement, while the dispersion of points reflects predictive accuracy. The high coefficients of determination (R² = 0.82–0.92) and low RMSE values confirm that the model reliably captured the multivariate response patterns of hydroponic maize fodder under different biochar treatments.


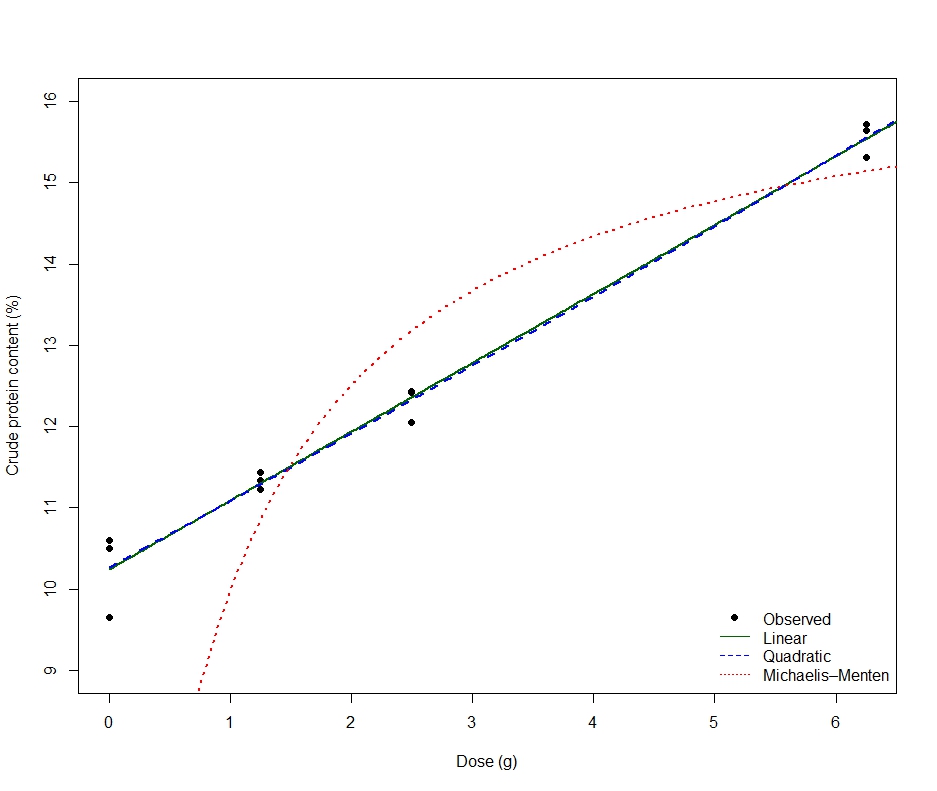


**Fig. S3.** Relationship between treatment dose and crude protein content (%), fitted using linear, quadratic, and Michaelis-Menten models.
